# National noncommunicable disease monitoring survey (NNMS) in India: Estimating risk factor prevalence in adult population

**DOI:** 10.1371/journal.pone.0246712

**Published:** 2021-03-02

**Authors:** Prashant Mathur, Vaitheeswaran Kulothungan, Sravya Leburu, Anand Krishnan, Himanshu Kumar Chaturvedi, Harshal Ramesh Salve, Ritvik Amarchand, Baridalyne Nongkynrih, P. Ganesh Kumar, Vinay Urs K. S., Lakshmy Ramakrishnan, A. Laxmaiah, Manjit Boruah, Sanjeev Kumar, Binod Kumar Patro, Pankaja Ravi Raghav, Prabu Rajkumar, P. Sankara Sarma, Rinku Sharma, Muralidhar Tambe, K. R. Thankappan, N. Arlappa, Tulika Goswami Mahanta, Rajnish P. Joshi, Neeti Rustagi, Sonia Gupta, Binod Kumar Behera, Sangita Chandrakant Shelke, Abhiruchi Galhotra, Pranab Jyoti Bhuyan, Abhijit P. Pakhare, Dewesh Kumar, Roshan K. Topno, Manoj Kumar Gupta, Atulkumar V. Trivedi, Suneela Garg

**Affiliations:** 1 Indian Council Medical Research–National Centre for Disease Informatics and Research, Bengaluru, Karnataka, India; 2 Centre for Community Medicine, All India Institute of Medical Sciences, New Delhi, India; 3 Indian Council Medical Research—National Institute of Medical Statistics, New Delhi, India; 4 Indian Council Medical Research—National Institute of Epidemiology, Chennai, Tamil Nadu, India; 5 Department of Cardiac Biochemistry, All India Institute of Medical Sciences, New Delhi, India; 6 Division of Community Studies, Indian Council Medical Research—National Institute of Nutrition, Hyderabad, Telangana, India; 7 Department of Community Medicine, Assam Medical College, Dibrugarh, Assam, India; 8 Department of Community and Family Medicine, All India Institute of Medical Sciences, Bhopal, Madhya Pradesh, India; 9 Department of Community and Family Medicine, All India Institute of Medical Sciences, Bhubaneshwar, Odisha, India; 10 Department of Community Medicine and Family Medicine, All India Institute of Medical Sciences, Jodhpur, Rajasthan, India; 11 Achutha Menon Centre for Health Science Studies, Sree Chitra Tirunal Institute for Medical Sciences and Technology, Thiruvananthapuram, Kerala, India; 12 Centre for Noncommunicable Diseases, National Centre for Disease Control, Directorate General of Health Services, New Delhi, India; 13 Department of Community Medicine, B J Govt. Medical College, Pune, Maharashtra, India; 14 Department of Public Health and Community Medicine, Central University Kerala, Kasaragod, Kerala, India; 15 Department of Community Medicine/Prevention & Social Medicine, Tezpur Medical College, Tezpur, Assam, India; 16 Department of Community and Family Medicine, All India Institute of Medical Sciences, Raipur, Chhattisgarh, India; 17 Regional Director Office, Ministry of Health and Family Welfare, Guwahati, Assam, India; 18 Department of Preventive and Social Medicine, Rajendra Institute of Medical Sciences, Ranchi, Jharkhand, India; 19 Department of Epidemiology, Indian Council Medical Research—Rajendra Memorial Research Institute of Medical Sciences, Patna, Bihar, India; 20 Department of Community Medicine, Government Medical College, Bhavnagar, Gujarat, India; 21 Department of Community Medicine, Maulana Azad Medical College and Associated Hospitals, New Delhi, India; Federal University of Pelotas, BRAZIL

## Abstract

**Background:**

The primary objective of National NCD monitoring survey (NNMS) was to generate national-level estimates of key NCD indicators identified in the national NCD monitoring framework. This paper describes survey study protocol and prevalence of risk factors among adults (18–69 years).

**Materials and methods:**

NNMS was a national level cross-sectional survey conducted during 2017–18. The estimated sample size was 12,000 households from 600 primary sampling units. One adult (18–69 years) per household was selected using the World Health Organization-KISH grid. The study tools were adapted from WHO-STEPwise approach to NCD risk factor surveillance, IDSP-NCD risk factor survey and WHO-Global adult tobacco survey. Total of 8/10 indicators of adult NCD risk factors according to national NCD disease monitoring framework was studied. This survey for the first time estimated dietary intake of salt intake of population at a national level from spot urine samples.

**Results:**

Total of 11139 households and 10659 adults completed the survey. Prevalence of tobacco and alcohol use was 32.8% (95% CI: 30.8–35.0) and 15.9% (95% CI: 14.2–17.7) respectively. More than one-third adults were physically inactive [41.3% (95% CI: 39.4–43.3)], majority [98.4% (95% CI: 97.8–98.8)] consumed less than 5 servings of fruits and / or vegetables per day and mean salt intake was 8 g/day (95% CI: 7.8–8.2). Proportion with raised blood pressure and raised blood glucose were 28.5% (95% CI: 27.0–30.1) and 9.3% (95% CI: 8.3–10.5) respectively. 12.8% (95% CI: 11.2–14.5) of adults (40–69 years) had ten-year CVD risk of ≥30% or with existing CVD.

**Conclusion:**

NNMS was the first comprehensive national survey providing relevant data to assess India’s progress towards targets in National NCD monitoring framework and NCD Action Plan. Established methodology and findings from survey would contribute to plan future state-based surveys and also frame policies for prevention and control of NCDs.

## 1. Introduction

In India, mortality from noncommunicable diseases (NCDs) accounted to 65% of total deaths in 2019 [1] and, over a period of 26 years (1990–2016) the burden of diseases has transitioned from communicable to noncommunicable diseases [2]. The total disability adjusted life years from NCDs was 30.5% in 1990 which increased in 2016 to 55.4% [2]. The population attributable fraction (PAF) for cardiovascular diseases and cancer due to smoking was 15.5% and 39.8% respectively. PAF for diabetes due to overweight and obesity was estimated to be 72.8% [3].

It was estimated that 1.3 million deaths in India could be prevented with tobacco control alone [2] and that a higher proportion of alcohol users belonged to the age group of 20–35 years [4–6]. Also, the population in India consumed twice the recommended daily salt [7], while, 86.7% consumed inadequate amount of fruits and vegetables; and only 47% consumed dark green and leafy vegetables per day [8, 9]. Studies [10] show that more than half the population in the country are physically inactive. The prevalence of overweight is predicted to increase by two-fold and obesity by three-fold by 2040 [11]. Results from the great India blood pressure survey showed 30.7% had hypertension [12]. Nearly 6.1 million deaths were accounted to NCDs in 2019, while 2.76% of deaths were from type 2 diabetes mellitus alone [1].

With a renewed focus to strengthen the multisectoral preventive and control strategies on NCDs, Government of India (GoI) launched the National Programme for Prevention and Control of Cancer, Diabetes, Cardiovascular Diseases (CVDs) and Stroke (NPCDCS) in 2010 and included the vertical cancer control programme. NPCDCS was brought under the National Health Mission in 2013 [13]. In accordance with the WHO-Global Voluntary NCD Targets and NCD Monitoring Framework [14], Ministry of Health and Family Welfare (MoHFW), GoI formulated the National NCD framework and action plan in 2013 [15]. Further in 2015, a comprehensive agenda of 17 Sustainable Development Goals (SDGs) and 169 targets by 2030 was adopted by the GoI [16]. These time bound commitments aim to reduce premature mortality from NCDs by one-third and achieve universal health coverage (UHC) [17, 18]. However, access to reliable information on disease magnitude, pattern, trends of exposures to NCDs risk factors and health system preparedness for NCDs form the prerequisites to policymakers in assessing status to frame suitable responses, monitor them and make midterm corrections [18].

The National NCD Monitoring Framework outlines 10 targets and 21 indicators to be achieved by 2025 [15]. Of the 21 indicators, ten are related to adults, three for adolescents, one for the households; and seven indicators are addressing the health system responses [15]. To measure the progress made in achieving these NCD targets by 2015, 2020 and 2025, the year 2010 served as baseline [15]. The data on risk factors mainly come from meta-analysis and reviews of smaller and ad-hoc surveys which have their own limitations [4–12]. The priority to monitor and evaluate the time bound national NCD targets needs a sustainable system of surveillance on NCDs, which was assessed as being inadequate.

Recognizing the need for establishing a robust system, the Indian Council of Medical Research (ICMR) was identified as the nodal agency for monitoring, evaluation and surveillance of the national NCD monitoring framework. ICMR-National Centre for Disease Informatics and Research (NCDIR), Bengaluru was tasked with the responsibility of conducting the first National NCD Monitoring Survey (NNMS). The primary objective was to generate national-level estimates of key NCD indicators (risk factors among adults and adolescents; and health systems response) identified in the national NCD monitoring framework. In addition, this effort would also create central and regional pool of resources to support the conduct of similar surveys at State/sub-national level in future, under the State NCD program.

This paper describes study protocols, methods and prevalence of NCD risk factors among adults aged between 18–69 years, which includes eight out of ten indicators for adults as per the national NCD monitoring framework [15] (Table 1).

**Table 1:** Targets and indicators for NCD prevention and control in India under the National NCD Monitoring Framework [15] for adults.

Framework element	Targets for 2025	No.	Indicators
	Outcomes		
**NCD Risk factors**			
Alcohol use	10% Relative reduction in alcohol use	1	Age standardised prevalence of current alcohol consumption in adults aged 18+ years.
Diabetes and obesity	Halt the rise in obesity and diabetes prevalence	2	Age standardised prevalence of obesity among adults aged 18+ years (defined as body mass index greater than 30 kg/m^2^)
		3	Age standardised prevalence of raised blood glucose/diabetes among adults aged 18+ years (defined as fasting plasma glucose value 126 mg/dl or on medication for raised blood glucose.
Physical inactivity	10% Relative reduction in prevalence of insufficient physical activity	4	Age standardised prevalence of insufficient physical activity in adults aged 18+ years (defined as less than 150 minutes of moderate-intensity activity per week, or equivalent).
Raised blood pressure	25% Relative reduction in prevalence of raised blood pressure	5	Age standardised prevalence of raised blood pressure among adults aged 18+ years.
Salt / sodium intake	30% Relative reduction in mean population intake of salt, with aim of achieving recommended level of less than 5g / day	6	Age standardised mean adult (aged 18+) population intake of salt per day.
Tobacco use	30% Relative reduction in prevalence of current tobacco use	7	Age standardised prevalence of current tobacco use (smoking and smokeless) among adults aged 18+ years.
	Additional indicator	8	Age standardised prevalence of adults (aged 18+ years) consuming less than 5 total servings (400 g) of fruit and vegetables per day.

*Out of 10 indicators for adults, two indicators are related to mortality and morbidity from NCDs, which have not been covered under NNMS 2017–18.

## 2. Materials and methods

The National NCD Monitoring Survey was a population-based cross-sectional survey conducted in 2017–18. A National Technical Working Group (TWG), Core group and research team of investigators of ten implementing agencies and collaborators were constituted for the survey. The survey agencies were those with considerable experience of field surveys and especially involving NCD risk factor surveys. All the study participants were enrolled only after obtaining written informed consent.

### 2.1 Sample size and sampling

The survey adopted stratified multistage sampling design using 2011 Census for India as a sampling frame covering age between 15–69 years (includes adolescents aged 15–17 years). Study population was divided into four subgroups/strata urban/rural and men/women (2 x 2). To estimate the key NCD risk factors for adults, the sample size was worked out using the 9% estimated prevalence of obesity reported from previous study [8], 15% relative precision, 95% confidence interval, 15% non-response rate and design effect of 1.5. The sample was enlarged to 12000 households as adolescents were to be enrolled from the same households. The national representative sample of 12000 households was equally allocated to both urban and rural areas, i.e. 6000 households each.

A sampling frame of all the districts with geographic contiguity was prepared and divided into 60 groups of districts with more or less equal population size using the 2011 Census data. Within each 60 groups, the primary sampling units (PSUs) (i.e. villages for rural and wards for urban) were arranged in ascending and descending order alternatively (implicit stratification) based on female literacy rates, proportion of agriculture workers. Thereafter that the rural and urban PSUs listed within each of 60 groups were separated for preparing the rural and urban frame of PSUs/Wards (urban).

At first stage, 300 PSUs from rural and 300 PSUs from urban sampling frame were selected using probability proportional to size (PPS) method sampling. In the second stage, one census enumeration block (CEB) was selected randomly from each ward in urban PSUs. In rural PSUs, if the village size was less than 50, the adjacent village was added so as to have adequate number of households. However, larger rural PSUs or villages with more than 400 households were divided into three or more segments so that an average size of each segment was about 150 to 200 households and, then two segments were selected randomly for household survey. The study teams mapped the selected CEBs or villages and listed all households within them. Following which 20 households were selected by circular systematic sampling method. From each household, in both rural and urban areas, one adult was selected using WHO-KISH method (STEPS) [19].

**Fig 1:**
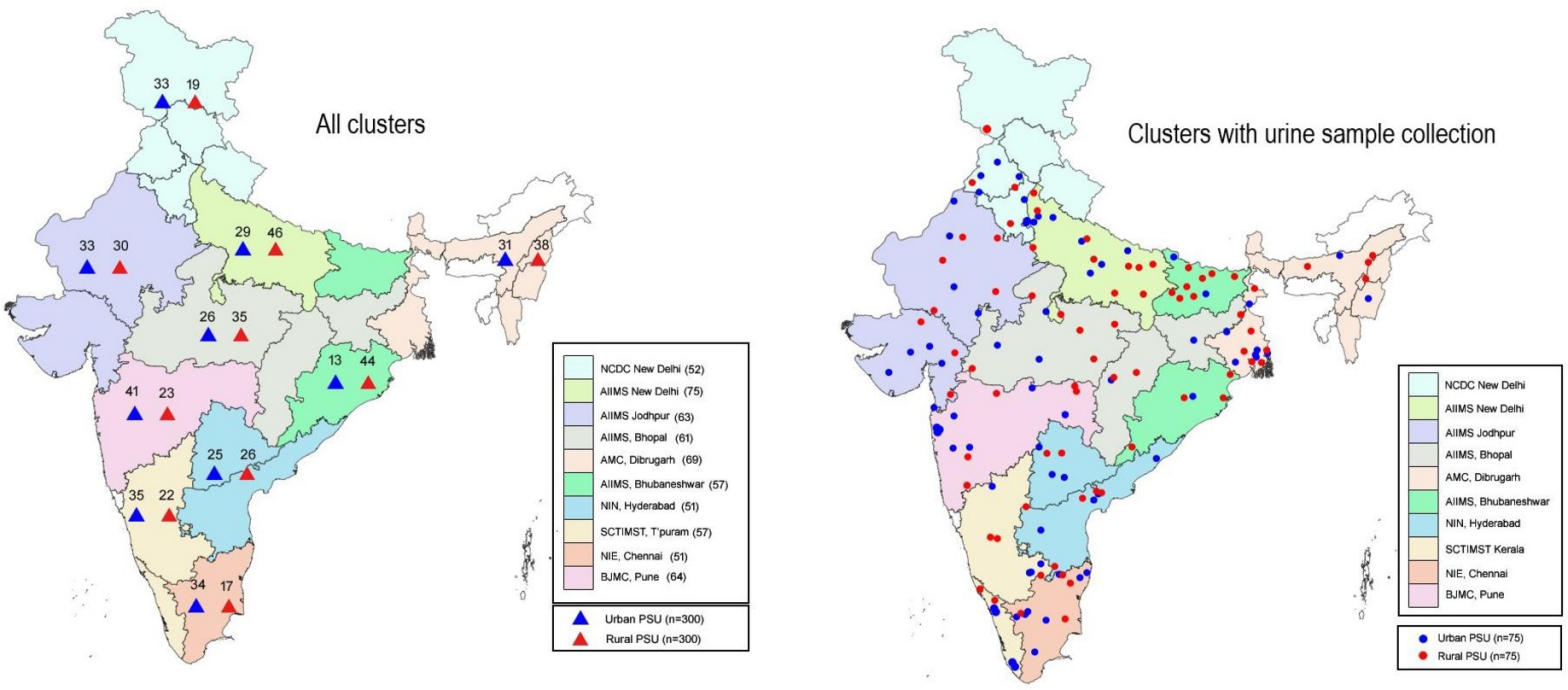
Geographical distribution of sampled clusters under NNMS 2017–18.

Based on a successful validation of use of spot urine sample prior to NNMS [20, 21], the estimation of salt intake was done using morning fasting urine sample in a sub-sample of subjects. Thus, a subsample of 3000 adults (18–69 years), representing 25% of the overall sample size, spread out across the country was selected. Total selected PSUs for the survey and urinary samples are mapped and shown in (Fig 1*)*.

### 2.2 Tools and data collection

The household and adult questionnaires were adapted from WHO-STEPwise approach to noncommunicable disease risk factor surveillance (WHO-STEPS) [22], Integrated disease surveillance project (IDSP)-NCD risk factor survey [8], and Global Adult Tobacco Survey-India (GATS) [23]. These were developed initially in English language and translated to eleven other Indian languages. Forward-backward translation method was followed for validation of the questionnaires and digitalized for data collection through an offline android-based application, Open Data Kit (ODK) [24, 25]. An instruction manual on ODK, an online web-based dashboard for reporting and monitoring of the survey events, and troubleshooting were also developed. All survey equipments were procured centrally for standardization, a standard operating procedure for use and calibration of equipment for measurements of height, weight, waist circumference, blood pressure, fasting blood glucose and urine sample collection was used.

Each field team of six members covered a cluster in five days and every implementing agency had two such teams working in parallel to undertake mapping and listing of PSUs, household and individual selection, household and individual interviews, health facility visits and biochemical sample collection through camps. Using prepared (villages) or procured (CEBs) location maps of PSU, teams prepared layout maps and undertook listing of structures, a starting point as determined randomly. Thereafter, every selected household was surveyed using the maps and addresses listed. Adult interview tracking sheets were used to interview the selected individual.

The household and adult interviews were done through face to face and information was collected in handheld devices in ODK software. The household form captured information on socio-economic status of the household (e.g. education level, occupation, housing type etc.), fuel use, cooking oil use and exposure to solid fuels by Head of Household. From the selected adult, in addition to demographic details on gender, age, education—If ever imparted in a school, or madrassa or gurukul or home schooled and occupation, information on behavioural risk factors like tobacco use (smoked and smokeless tobacco; second hand tobacco smoke exposures) and alcohol consumption (quantity, frequency and patterns), diet (fruits and vegetables intake—servings and frequency and knowledge, attitude and practices of dietary salt intake) and details on moderate and vigorous physical activity at workplace or home, during travel and during leisure were assessed using Global Physical Activity Questionnaire were obtained. Information on diagnostic and treatment history, screening, consultation and treatment services for diabetes, hypertension, raised cholesterol and cardiovascular diseases were collected. Question by question details have been provided in S1 Table.

The physical measurements of height, weight and waist circumference (WC) were measured using standardised equipment—portable stadiometer (SECA 213), digital weighing scale (SECA 803) and measuring or tension tape (SECA, 201) recommended by WHO-STEPS (*Seca Gmbh Co*, *Hamburg*, *Germany)*. Both height and weight of consented participants were measured while in barefoot and with light clothing, ensured body was erect, head looking straight facing interviewer and arms on the side. The measurements of weight in Kilograms divided by height in metres squared determined the Body Mass index. The WC was measured at the end of a normal expiration, with arms relaxed at the sides, keeping reference of mid-axillary line, a midpoint of lower part of the lowest rib and the highest point of the hip was marked to measure to the nearest 0.1cm. Blood pressure was measured using standard automatic blood pressure machine *(OMRON HEM–7120*, *Omron corporation*, *Kyoto*, *Japan)*. Three blood pressure measurements were recorded in a seated position with three minutes resting interval between the measurements, an average of last two readings was considered. All instruments were regularly calibrated and calibration log books were maintained by the survey teams.

A camp was organized for glucometer-based *(Gluco spark*, *Sensa core*, *Telangana*, *India)* capillary fasting blood glucose (dry strip method) testing and spot urine sample collection. All participants were given urine collection bottles, appointment slips and instruction leaflet on fasting and urine collection one day before the camp. The collected urine bottles were stored in aliquots and transported in cold chain to pre-designated facilities for storage at -20^0^ C. The stored urine samples were couriered from all centres in cold storage and tested in batches of 80–100 urine samples at the central reference laboratory at AIIMS Delhi. The urinary Na, K levels were estimated in the automated analyzers *(AU680 Chemistry analyzer*, *Beckman Coulter*, *CA*, *USA)* using indirect Ion-Selective Electrode (ISE) method. Urinary Creatinine levels were measured by Jaffe’s method on Roche analyzer *(P800 Modular Analytics*, *Roche Diagnostics*, *Mannheim*, *Germany)* using a commercially available kit *(Ref*. *11875418–216*, *Roche diagnostics*, *Germany)*.

In situation when households were found locked or selected respondent was not available during the first visit, a notification of visit was left. A minimum of 3 additional visits were made which suited the time given by the participants. In case of refuse/partially completed forms, the response status decision was confirmed by the team leader, followed by research scientist and Co-PI or PI.

### 2.3 Standardization and quality control

Trainings were conducted at national level as Training of Trainers model followed by training of the field team jointly by central and regional team. A mock pilot survey was undertaken by each implementing agency following the regional training in 2 non-selected PSUs. In addition, specific re-training sessions were conducted after 1–2 months of the initiation of field activities to refresh survey procedures and clarify any issues confronted in the field. To ensure quality control, supervision mechanisms were built-in at various phases of survey implementation involving local investigators, Core group members and National TWG experts.

### 2.4 Ethical approval

The study was approved by National Centre for Disease Informatics and Research Institutional Ethics Committee of the coordinating centre, the Indian Council of Medical Research—National Centre for Disease Informatics and Research, Bengaluru—NCDIR/IEC/2017/4 dated on 03.02.2017 followed by all other implementing agencies from their respective IECs (Institute Ethics Committee, All India Institute of Medical Sciences, New Delhi—IEC-213/05.05.2017, RP-30/2017 dated 23-05-2017; NCDC Ethics Review Committee (NERC), National Centre for Disease Control, New Delhi—2017/NERC/11 dated 26-09-2017; All India Institute of Medical Sciences, Jodhpur–Institutional Ethics Committee—AIIMS/IEC/2017/283 dated 24-05-2017; All India Institute of Medical Sciences, Bhopal—Institutional Human Ethics Committee—IHEC-LOP/2017/EF0047 dated 07-07-2017; Institutional Ethics Committee (H), Assam Medical College, Dibrugarh—AMC/EC/2693 dated 22-12-2017; All India Institute of Medical Sciences, Bhubaneshwar—Institutional Ethics Committee—T/EM-F/CMFM/17/08 dated 15-05-2017; Institutional Ethics Committee, B J G Medical College and Sassoon General Hospitals, Pune—BJGMC/IEC/Pharmac/ND-ICMR 0717101–101 dated 25-07-2017; Institutional Ethics Committee, National Institute of Nutrition (ICMR), Hyderabad—ECR/351/Inst/AP/2015 dated 10-01-2018, Sree Chitra Tirunal Institute for Medical Sciences and Technology, Trivandrum—Institutional Ethics Committee—SCT/IEC/1057/May-2017 dated 13-06-2017 and Institutional Human Ethics Committee–National Institute of Epidemiology–Indian Council of Medical Research—NIE/IHEC/201607-02 dated 29-07-2016). All the study participants were enrolled only after obtaining written informed consent and those who were detected with risk factors or the presence of disease were referred to the nearest public health facility for further evaluation and management. The required support and concurrence of local government agencies in implementing the survey were obtained. Health promotion pamphlets in local language were provided to all participants irrespective of their health status.

### 2.5 Definitions used

Standard definitions were used for estimating all behavioural and biological indicators (tobacco use, alcohol use, diet, physical activity, BMI, raised blood glucose and blood pressure) [26]. WHO-ISH Cardiovascular disease (CVD) risk prediction charts, were used for arriving at ≥30% ten-year cardiovascular risk estimates for adults aged 40–69 years [27] (Table 2). Mean salt intake of the population was estimated by applying the Intersalt equation [7, 21, 28–30] and it was multiplied by 2.54 to arrive at values in grams/day from the estimated sodium, potassium and Creatinine excretion.

**Table 2:** List of indicator definitions used in the National NCD Monitoring Survey (NNMS) 2017–18.

Indicator	Definition
**Current tobacco use**	Use of any form of tobacco (smoke or smokeless) in the last 12 months preceding the survey.
**Current alcohol use**	Consumption of alcohol in the last 12 months preceding the survey.
**Heavy episodic drinking**	Those engaged in consuming six or more standard drinks (60 grams) in a single drinking occasion over the past 30 days.
**Inadequate consumption of fruits and / or vegetables**	Percentage of subjects who ate less than five servings of fruit and/or vegetables on an average per day.
**INTERSALT equation with Potassium**	**Men:** 23 × (25.46 + [0.46 × spot Na (mmol/L)]–[2.75 × spot Cr (mmol/L)]—[0.13 × spot K(mmol/L)] + [4.10 × BMI (Kg/m^2^)] + [0.26 × age (years)]) **Women:** 23 × (5.07 + [0.34 × spot Na (mmol/L)]–[2.16 × spot Cr (mmol/L)]—[0.09 × spot K(mmol/L)] + [2.39 × BMI (Kg/m^2^)] + [2.35 × age (years)] − [0.03 × age^2^ (years)])
**Insufficient physical activity in adults**	Percentage of adults aged 18–69 years who spent <150 minutes of moderate-intensity physical activity per week OR <75 minutes of vigorous-intensity physical activity per week OR an equivalent combination of moderate and vigorous intensity physical activity accumulating <600 MET minutes per week.
**Overweight and obesity**	Overweight was BMI 25.0–29.9 Kg/m^2^ and obesity was ≥ 30.0 Kg/m^2^ as per WHO cut-off.
**Central obesity**	Waist circumference of ≥90cm in males and ≥80cm in females (as per South Asia Pacific Guidelines).
**Raised blood pressure**	Raised blood pressure (including those on medication) in adults aged between 18–69 years with a systolic blood pressure ≥140 mm of Hg and/or diastolic blood pressure ≥90 mm of Hg including those on medication for raised blood pressure.
**Raised blood glucose**	Raised blood glucose (including those on medication) in adults aged 18–69 years with fasting blood glucose value ≥126 mg/dl including those on medication for raised blood glucose.
**Clustering of risk factors**	Presence of ≥3 risk factors; daily tobacco use, inadequate fruits and/or vegetable intake, insufficient physical activity, overweight (BMI ≥25.0Kg/m^2^), raised blood pressure and raised fasting blood glucose including those on medication among adults aged 18–69 years.
**Ten-year CVD risk of ≥30%**	Defined as per WHO/ISH prediction charts for CVD risk for South-East Asia Region, according to the age (40–69 years), gender, systolic blood pressure, current smoked tobacco use and diabetes (previously diagnosed/fasting blood glucose concentration ≥126 mg/dl).

### 2.6 Data management and statistical analysis

The global positioning system was used to map the co-ordinates of the participating households and adults at the end of each interview. The data collected was saved in a draft format and once validated and finalized they were synced to the secure central server. Data was cleaned in SPSS Version 22.0 and weighted to provide prevalence estimates at the population level, households, area of residence (urban and rural), individuals for age group and gender. Final weighted data were analysed in STATA 14.1 by complex survey analysis [31, 32] and the results are presented by descriptive statistics in mean and proportion with 95% confidence intervals (CIs).

## 3. Results

**Fig 2:**
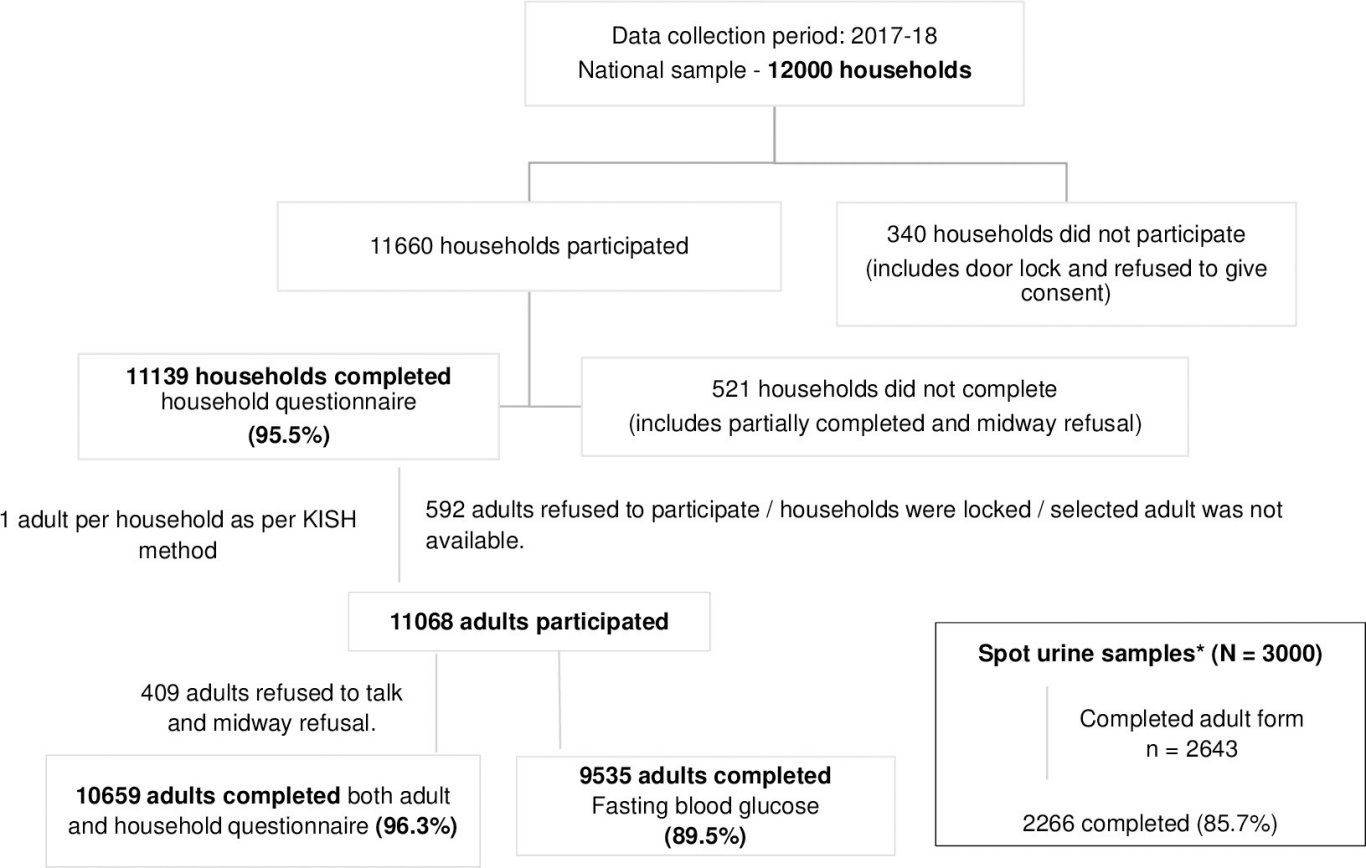
Response rate at household and individual level of components in the survey.

The overall response rate of the survey was 96.3% (Fig 2). From, a subsample of 3000 a total of 2266 (85.7%) participants provided spot urine samples.

### 3.1 Sociodemographic characteristics

In total, 10659 adults aged between 18–69 years participated in the survey of which 5048 (47.4%) adults were from urban and 5611 (52.6%) adults belonged to rural areas. The mean age was 40.1 (± 13.8) years, with a higher preponderance of women (n = 5825; 54.6%). Majority had formal schooling which included being imparted education ever either in school or madrassa or gurukul (71.2%) and were married (79.4%). Most men were unskilled manual labours (34.0%), and the majority of women were homemakers (66.9%). (Table 3)

**Table 3:** Socio demographic characteristics of participants aged 18–69 years included in the study.

Socio demographic characteristics	Men		Women		Urban		Rural	
	n	% (95% CI)	n	% (95% CI)	n	% (95% CI)	n	% (95% CI)
**Age group (in years)**								
18–44	2986	70.2 (68.0–72.3)	3643	69.1 (67.2–71.0)	3127	68.2 (65.1–71.2)	3502	70.4 (68.3–72.4)
45–69	1848	29.8 (27.7–32.0)	2182	30.9 (29.0–32.8)	1921	31.8 (28.8–34.9)	2109	29.6 (27.6–31.7)
**Education Status**								
No formal education	816	17.1 (15.1–19.3)	2215	41.9 (39.1–44.8)	898	14.9 (12.7–17.5)	2133	36.1 (33.6–38.7)
Formal education	4018	82.9 (80.7–84.9)	3610	58.1 (55.2–60.9)	4150	85.1 (82.5–87.3)	3478	63.9 (61.3–66.4)
Highest level of education*								
Less than Class 6	794	21.3 (19.0–23.9)	862	26.3 (23.3–29.4)	644	14.8 (12.7–17.2)	1012	28.9 (25.8–32.2)
6 to 8	700	18.1 (16.3–20.0)	742	21.9 (19.8–24.1)	678	15.7 (13.9–17.7)	764	22.2 (20.4–24.1)
8 to 12	1634	41.3 (38.5–44.0)	1371	37.1 (34.1–40.3)	1680	42.0 (38.8–45.2)	1325	38.0 (34.9–41.3)
Degree and higher	898	19.3 (17.3–21.6)	661	14.7 (12.7–17.0)	1163	27.5 (24.2–31.0)	396	10.9 (9.3–12.7)
Occupation status*								
Employed	3985	84.1 (82.5–85.5)	1418	26.8 (23.7–30.0)	2423	55.3 (52.4–58.2)	2980	57.1 (54.5–59.7)
Unemployed	808	15.2 (13.9–16.6)	351	6.3 (5.3–7.5)	632	13.3 (11.7–14.9)	527	9.8 (8.7–11.0)
Home maker	34	0.7 (0.4–1.2)	4049	66.9 (63.6–70.1)	1986	31.4 (28.7–34.3)	2097	33.1 (30.6–35.7)

n = unweighted number; % = weighted percentage

*excludes “no response”.

### 3.2 Behavioural and metabolic risk factors

The prevalence of current tobacco use (either smoked or smokeless) was 32.8% (95% CI (30.8–35.0). Its use was higher in adults residing in the rural areas [Rural: 36.8% (95% CI: 34.3–39.2); urban: 25.1% (95% CI: 21.5–29.2)] and among men [51.2% (95% CI: 48.3–54.1)]. However, the prevalence of its use among women was 13.0% (95% CI: 11.1–15.2). Overall current smokeless tobacco use [24.7% (95% CI: 22.7–26.9)] was more prevalent than smoking [12.6% (95%CI: 11.3–14.1)]. The mean age of tobacco initiation was 21.1 (95% CI: 20.6–21.6) years. Overall, 28.0% of adults used one or other form of tobacco daily, with a higher proportion of users from the rural areas [31.7% (95% CI: 29.5–34.0) than urban [20.5% (95% CI: 17.0–24.4)]. Higher percentage of adults from rural areas (Rural: 8.6%; urban:4.9%) and men (Men: 13.6%; women: 0.7%) smoked bidis, while those from urban areas smoked cigarettes daily (urban: 4.2%; rural: 1.6%). Among those adults [35.8% (95% CI: 31.7–40.2)] who attempted to quit the habit of smoking in past year, women were in higher proportion (men: 35.2%; women: 46.8%) (Table 4 and S1 Table).

**Table 4:** NCD risk factors (behavioural and metabolic) among adults (18–69 years).

Prevalence of risk factors (18–69 years)	Men		Women		Urban		Rural		Overall	
	n	% (95% CI)	n	% (95% CI)	n	% (95% CI)	n	% (95% CI)	n	% (95% CI)
**Behavioral risk factors**										
**Tobacco use (%)**										
Current tobacco use (Smoked or smokeless tobacco)	2354	51.2 (48.3–54.1)	685	13.0 (11.1–15.2)	1172	25.1 (21.5–29.2)	1867	36.8 (34.3–39.2)	3039	32.8 (30.8–35.0)
Current smokeless tobacco use	1640	36.5 (33.6–39.5)	633	12.0 (10.2–14.1)	840	17.6 (14.6–20.9)	1433	28.3 (25.8–31.0)	2273	24.7 (22.7–26.9)
Current smoked tobacco use	1081	23.0 (20.8–25.5)	67	1.3 (0.9–2.0)	496	11.6 (9.2–14.6)	652	13.1 (11.6–14.8)	1148	12.6 (11.3–14.1)
Smokers who attempted to quit the habit in past year	378	35.2 (31.0–39.7)	27	46.8 (29.4–65.1)	181	38.3 (32.7–44.2)	224	34.7 (29.3–40.5)	405	35.8 (31.7–40.2)
**Second-hand tobacco smoke exposure in past 30 days (%)**										
At home	1659	36.1 (33.2–39.1)	1517	27.7 (25.0–30.5)	1340	25.8 (21.9–30.2)	1836	35.2 (32.5–38.0)	3176	32.1 (29.7–34.5)
During travel or at workplace	2364	48.7 (45.5–51.9)	1398	23.3 (20.5–26.2)	1950	39.6 (34.4–45.0)	1812	34.9 (31.9–38.1)	3762	36.5 (33.8–39.3)
**Alcohol use (%)**										
Current alcohol use	1315	28.3 (25.8–31.0)	95	2.4 (1.5–3.9)	655	14.2 (12.5–16.0)	755	16.7 (14.4–19.3)	1410	15.9 (14.2–17.7)
Heavy episodic drinking	507	10.9 (9.5–12.6)	16	0.5 (0.2–1.3)	251	5.7 (4.7–6.8)	272	6.1 (4.9–7.5)	523	5.9 (5.1–6.9)
Lifetime abstainers	3226	66.1 (63.2–68.9)	5696	97.3 (95.8–98.3)	4243	82.8 (80.7–84.8)	4679	80.2 (77.5–82.7)	8922	81.1 (79.2–82.9)
**Dietary Practices**										
Inadequate consumption of fruits and / or vegetables (%)	4714	98.0 (97.1–98.6)	5732	98.8 (98.3–99.2)	4911	97.7 (96.7–98.5)	5535	98.7 (97.9–99.2)	10446	98.4 (97.8–98.8)
Mean population salt intake (g/day) (Mean)	1081	8.9 (8.7–9.2)	1185	7.1 (6.9–7.2)	1025	8.3 (8.0–8.7)	1241	8.0 (7.8–8.2)	2266	8.0 (7.8–8.2)
**Physical activity (%)**										
Insufficient physical activity	1642	30.9 (28.3–33.7)	3170	52.4 (50.0–54.7)	2628	51.7 (48.6–54.8)	2184	36.1 (33.9–38.3)	4812	41.3 (39.4–43.3)
**Metabolic risk factors (%)**										
Overweight (BMI ≥ 25.0 Kg/m^2^)	1259	23.3 (21.0–25.7)	1991	29.3 (27.0–31.7)	2119	42.5 (39.8–45.2)	1131	18.0 (16.1–20.1)	3250	26.1 (24.2–28.1)
Obesity (BMI ≥ 30.0)	264	4.3 (3.6–5.2)	613	8.3 (7.1–9.7)	629	11.2 (9.6–13.0)	248	3.7 (3.1–4.6)	877	6.2 (5.5–7.0)
Raise blood pressure	1504	29.9 (27.9–32.0)	1738	27.0 (25.2–28.8)	1726	34.0 (32.0–36.1)	1516	25.7 (23.8–27.8)	3242	28.5 (27.0–30.1)
Raised blood glucose	489	8.5 (7.4–9.7)	660	10.2 (8.8–11.8)	720	14.4 (12.5–16.4)	429	6.9 (5.7–8.3)	1149	9.3 (8.3–10.5)

*n = unweighted number; % = weighted percentage*.

Overall, 81.1% of adults had reported to be lifetime abstainers of alcohol, with a higher proportion being women (97.3%) than men (66.1%). However, a larger proportion of men aged 18–69 years had engaged in alcohol use ever in life [33.9% (95% CI: 31.1–36.8)] and heavy episodic drinking [10.9% (95% CI: 9.5–12.6)] than women (0.5%). Amongst adults who ever consumed alcohol, a higher proportion of men abstained from drinking in the last 12 months (men: 16.4%; women: 9.4%). The prevalence of alcohol use in last 12 months was also higher among men [28.3% (95% CI: 25.8–31.0)] than women (2.4%). The mean age of initiation of alcohol use [22.2 (95% CI: 21.6–22.7)] years and age of initiation of tobacco (21.1 years) were similar (Table 4 and S1 Table).

The average reported consumption of fruits and/or vegetables was 2 servings per day [1.7 (95% CI: 1.6–1.8)], which was much less than the WHO recommendations. Men consumed more salt [8.9 (95% CI: 8.7–9.2)] g/day (women: 7.1 g/day) and always or often added extra salt right before eating food [men: 16.4% (95% CI: 14.4–18.6); women: 13.7%)]. Higher proportion of men (69.7%) than women (58.4%) thought lowering salt consumption is important and took steps to reduce salt intake. More than half the percentage of urban adults [51.7% (95% CI: 48.6–54.8)] and women [52.4% (95% CI: 50.0–54.7)] were physically inactive compared to rural adults (36.1%) and men (30.9%) respectively. (Table 4 and S2 Table).

Overall prevalence of overweight and obesity among adults was 26.1% (95% CI: 24.2–28.1) and 6.2% (95% CI: 5.5–7.0) respectively. A higher percentage of adults in urban areas (urban: 42.5%; rural: 18.0%) and women (men: 23.3%; women: 29.3%) were overweight, obese (urban: 11.2%; rural: 3.7% and men: 4.3%; women: 8.3%) and with central obesity (urban: 48.2%; rural: 24.2% and men: 24.4%; women: 40.7%) when compared to their respective sub-groups. Higher prevalence of raised blood pressure [urban: 34.0% (95% CI: 32.0–36.1); rural: 25.7%)] and raised blood glucose [urban: 14.4% (95% CI: 12.5–16.4); rural: 6.9%)] was seen among adults residing in urban areas. However, gender distribution showed raised blood pressure was higher among men [men: 29.9% (95% CI: 27.9–32.0); women: 27.0%)], while the prevalence of raised blood glucose was high among women [women: 10.2% (95% CI: 8.8–11.8); men: 8.5%)] (Table 4 and S1 Table).

Two-fifth [40.2% (95% CI: 38.5–42.0)] of the adults 18–69 years had clustering of more than or equal to 3 risk factors (daily tobacco use, inadequate fruits and/or vegetable intake, insufficient physical activity, overweight (BMI ≥25.0 Kg/m^2^), raised blood pressure and raised fasting blood glucose including those on medication among adults aged 18–69 years) and 12.8% (95% CI: 11.2–14.5) of adults 40–69 years had ≥30% ten-year CVD risk or reported history of CVD with a higher proportion from urban areas (Table 5).

**Table 5:** Composite risk assessment among adults (18–69 years).

	Men		Women		Urban		Rural		Overall	
	n	% (95% CI)	n	% (95% CI)	n	% (95% CI)	n	% (95% CI)	n	% (95% CI)
**Clustering of risk factors (18–69 years)**										
≥ 3 risk factors	1952	**41.4** (39.1–43.7)	2211	**39.0** (36.8–41.1)	2303	**52.8** (50.5–55.1)	1860	**34.2** (32.3–36.2)	4163	**40.2** (38.5–42.0)
**10-year CVD risk (40–69 years)**										
< 10%	1057	**71.9** (68.5–75.1)	1133	**67.2** (63.7–70.4)	1075	**68.5** (65.0–71.9)	1115	**70.3** (67.1–73.3)	2190	**69.6** (67.3–71.9)
10 to 20%	160	**9.1** (7.4–11.2)	218	**12.4** (10.6–14.4)	181	**9.7** (8.0–11.8)	197	**11.3** (9.6–13.3)	378	**10.7** (9.4–12.1)
20 to 30%	93	**5.8** (4.5–7.5)	151	**8.1** (6.6–9.9)	133	**8.4** (6.8–10.3)	111	**6.0** (4.8–7.4)	244	**6.9** (5.9–8.0)
≥ 30% or with existing CVD	235	**13.2** (11.2–15.5)	206	**12.3** (10.0–15.1)	242	**13.4** (11.1–16.0)	199	**12.4** (10.4–14.8)	441	**12.8** (11.2–14.5)

n = unweighted number; % = weighted percentage.

## 4. Discussion

NNMS is the first comprehensive fully digitalized national level survey that provides key estimates on NCD risk factors according to the National NCD framework and action plan of India. It makes available a compilation of study tools and protocols developed to meet global and national standards and establish sustainable mechanisms to undertake state or district level surveys. The strengths of the survey are its representativeness, standardized approach in collaboration with well reputed implementation agencies, high response rates, and rigorous quality control procedures. This survey was built on similar surveys done between 2003–2008 across India by most of this investigator group.

NNMS results on sociodemographic characteristics was comparable to the demographic data from Indian Census 2011. However, a slightly higher proportion were literate (71.2%) than census data (68.9%), this could be due to different data collection time periods [33].

This survey found use of tobacco and alcohol to be most prevalent among men between 18–69 years, which was consistent with other survey findings [34–36]. However, prevalence of current tobacco use was slightly higher (NNMS: 32.8%) than that reported from GATS– 2-year 2016–17 (28.6%) [34], and National Mental Health Survey (NMHS) in year 2016 (25%) which had much higher sample size [36]. The reasons for inconsistencies may be from different study objectives and design, sampling strategy, coverage, age groups selected, weighting procedures, questionnaires adopted, and methodology, or emerging use of smokeless tobacco [NNMS: 24.7% (95% CI: 22.7–26.9)] compared to the GATS-2 (21.4%) [34]. But, the high prevalence of current smokeless tobacco use among men and adults in rural areas was consistent with findings from GATS-2 [34]. The prevalence of current alcohol use was 15.9% (NNMS) and this was comparable to the results from the report on magnitude of substance use in India– 2019 (14.6%) [35].

Prevalence of physical inactivity and overweight were found to be 41.3% and 26.1% respectively. These findings were comparable to the recent [37] meta-analysis [34% (95% CI: 22.3–47.7)] and results (54.1%) of Anjana RM et al from the ICMR-INDIAB (phase-I) survey [10]. The proportion of adults who are overweight was 26.1% and this was comparable to the projection estimates of 12.9% in 2005 to 27.8% by 2030 from Kelly T et al [38, 39] in 2008. Also, the study estimated an expected rise in prevalence of obesity from 4.0% in 2005 to 5.0% by 2030 [38, 39] and the findings from NNMS 2017–18 reveals and substantiates the high prevalence in obesity (6.2%).

The results on the prevalence of raised blood pressure (28.5%) and raised blood glucose (9.3%) were comparable to the results from population-based studies, ICMR-INDIAB study and WHO report 2015. [12, 40–46] The Global Burden of Disease study (Indian estimates) reports blood pressure as one of the three leading risk factors for national disease burden [2]. Excess intake of salt is a well-established risk factor for high BP [45]. The mean intake of salt at the population level was found to be 8 gm/day which is comparable to the meta-analysis by Petersen et al, reporting salt consumption in India to be more than the WHO recommended maximum of 5g/day [22]. This was for the first time a national level salt estimation was done using spot urine samples.

Majority (98.4%) of adults aged between 18–69 years consumed inadequate fruits and vegetables and this finding was similar to the STEPs survey done in Punjab by Thakur et al [47]. Also, the results on the mean servings of fruits and /vegetables consumed per day were similar to those from STEPs survey done in Kerala by Sarma et al [48].

More than or equal to 30% of 10-year cardiovascular risk prediction for the study participants aged 40–69 years using WHO-ISH CVD risk prediction charts [27] were estimated to be 12.8%. Similar results were observed in other studies done within the country [49, 50]. Furthermore, compared to the 17.7%, for men and 24.7% women who had clustering of risk factors from the study by Ahmed et al study [51], NNMS showed 41.4% in men and 39.0% in women showed clustering of more than or equal to 3 risk factors, this difference could be attributed to the methodology and the inclusion of risk factors to arrive at the clustering.

Government of India has long instituted measures to prevent and control NCD risk factors in the country by several programmes. The presence of NCD risk factors at high proportions in adult residing in urban and rural areas calls for stepping up of responses on priorities for immediate and future policy making. The study reveals and highlights the indicators that need considerable approaches to reinforce the already existing policies and programmes [52, 53].

We recommend regularization of similar large comprehensive NCD risk factor surveys like NNMS every three to four years. There is a need to replace and harmonize vertical single risk factor surveys, setting up of national NCD surveillance units under Ministry of Health and Family Welfare, Government of India to primarily gather, analyse and report timely data for action and closely liaison with the stakeholders for interventions periodically. Timely dissemination of survey results through multisectoral engagement, linkages for collective action and community empowerment are crucial. Further, it is essential to undertake future State level surveys for studying variations within the country and arrive at an appropriate NCD research agenda which is relevant to the local needs.

In addition to the main strengths of the survey already addressed, we used spot urine samples to estimate urinary sodium and arrive at national level mean salt intake among the adult population on a well representative sample which was done for first time.

The cross-sectional nature of survey limits causality relation and provides only burden of risk factors at a point of time. There might be over or under reporting related to tobacco, alcohol, physical activity and diet due to social desirability bias. However, the major limitations of the survey include challenges in arriving at State-based estimates. However, the large sample size and weighted proportions at national level are conclusive.

## 5. Conclusion

The survey results form a baseline for the country to monitor the NCD targets devised by global NCD framework to be achieved by 2025.

The partnerships and collaborators involved in implementation of NNMS 2017–18 have yielded many noticeable strengths and learnings for the way forward. NNMS was specifically designed to enable monitoring the National NCD monitoring framework at for which data from other sources a national level were not available. It provides standardised tools and methods for future NCD surveillance activities at national/sub-national levels to enable measuring population level trends and impact of NCDs and interventions to tackle them. Periodicity of such surveys through committed funding and mandate would assess further progress against it over time for planning interventions and accelerate actions to achieve its targets.

## Supporting information

S1 File Survey questionnaire for adults (18–69 years). (PDF)

S1 Table NCD risk factors (behavioural and metabolic) among adults (18–69 years). (DOCX)

S2 Table NCD risk factors among adults aged between 18–69 years (mean). (DOCX)
