# Supplementary material for: National noncommunicable disease monitoring survey (NNMS) in India: Estimating risk factor prevalence in adult population
Source: PLoS One. 2021 Mar 2;16(3):e0246712. doi: 10.1371/journal.pone.0246712 (PMC7924800; doi:10.1371/journal.pone.0246712)
Supplement: S1 File — (PDF) [file pone.0246712.s001.pdf]

Individual ID | | | | | | | |

National Non-Communicable Disease Monitoring Survey - India Risk Factor Survey, 2017

Adult Individual (18-69 years) Interview Schedule

**I 1**	Enter interviewer ID
**I 2**	Scan the barcode for the Selected Household
**I 2**	Enter the barcode for the Selected Household
	Agency Code:
	PSU Code:
	House Hold Number:
	Introduce yourself and tell the purpose of the visit:<br>I am _(name)_____ from _(institutional agency)_____ . We had visited your house yesterday and had selected you as one of the participant for this national survey.
**I 3**	Status<br><br>Locked =1<br>Asked to come later=2<br>Refused=3<br>Ready to talk=4
**I 4**	Name of the selected adult (Fill this from the adult tracking sheet)
**I 5**	Consent has been read and obtained<br>Yes =1 ; No=2
**I 6**	Interview language
**I 7**	Contact phone number wherever possible

**Section 1.A**		**Demographic Information**		
Questions		Response		Skip
C 1	Sex of the respondent<br>**उत्तरदाता का लिंग**	Male पुरुष ...1<br>Female स्त्री ...2	☐	

Individual ID | | | | | | | |

	Write the sex of the individual as observed			
C 2	What is your date of birth? आपकी जन्म तिथि क्या है ? Record the date of birth of the person. If unknown or do not respond.	Fill 77 77 7777 if do not remember	D D M M Y Y Y Y	*If date provided go toC4यदि तिथि बतायी तो C4 पर जाएँ*
C 3	What is your age? आपकी उम्र कितनी है Record the age of the person. If unknown help him/her estimate the age by asking further questions on well known major events or can ask the parents or guardian.	Age in completed years पूर्ण हुए सालों में उम्र		
C 4	Which religion do you belong to? आप किस धर्म को मानते हैं Select the appropriate option	Hinduism हिन्दू .....01<br>Islam इस्लाम ....02<br>Christianity इसाई ....03<br>Sikhism सिख ....04<br>Buddhism बौध ...05<br>Jainism जैन ....06<br>Do not know .....07<br>None कोई भी नहीं ....11<br>Others(specify) अन्य (स्पष्ट करें)....12	अन्य (specify ________ ____/स्पष्ट करें)	

C 5	Which social group do you belong to? (as per state govt. notification) **आप किस जाति समुदाय से सम्बंधित हैं ?** Select the appropriate response.	General जनरल ....1<br>OBC ओ बी सी ....2<br>Schedule caste अनुसूचित जाती ....3<br>Schedule tribe अनुसूचित जनजाति ....4<br>Don't know नहीं जानते .....7<br>No response कोई जवाब नहीं .....8<br>Cannot decide (Specify) 9		
C 6	What is your current marital status? **आपकी वर्तमान वैवाहिक स्थिति क्या है** Select the appropriate response.	Never married कभी शादी नहीं हुई .... 1<br>Living in /co habiting साथ रह रहे हैं ....2<br>Currently Married (Including non-cohabiting) शादीशुदा हैं(साथ नहीं रह रहे भी शामिल) ....3<br>Not living together/Separated साथ नहीं रह रहे /अलग हो चुके हैं .....4<br>Divorced तलाकशुदा ....5<br>Widowed विधवा या विधुर ...6<br>Do not know ...7<br>No response/Don't want to tell कोई जवाब नहीं 8		
C 7	Have you ever been imparted any education? **क्या आपने कभी शिक्षा प्राप्त की है ?** Select the appropriate response.	No......**नहीं** ....1<br>Yes in a formal school......हाँ **औपचारिक** स्कूल में....2<br>Yes in a madrassa/ Gurukul etc......हाँ **मद्दरसे**/गुरुकुल इतियादी में....3<br>Yes home schooled हाँ **घर से पढाई की** में...4		*If No go to C9 यदि नहीं तो C9 पर जाएँ*
C 8	If yes, what is the highest level of education you completed? **यदि हाँ तो आपने सर्वोच्च**	Less than Class 6... क्लास 6 से कम.... 01<br>Class 6or 7 or 8 completed ... क्लास 6या 7 या 8 की शिक्षा पूरी......02		

Individual ID | | | | | | | |

	शिक्षा कहाँ तक ली है ? Choose the years of education even if it was not gained in a formal school .e.g. a person who completed 6 years of Madrassa education or 6 years of education in a school or 6 years of education at home all will be coded as "02"	Class 9 or 10 completed...क्लास 9 या 10की शिक्षा पूरी...03<br>Class 11 or 12completed.. क्लास 11 या 12 की शिक्षा पूरी...04<br>Graduation or diploma completed...स्नातक स्तरया डिपलोमा पूरा ....05<br>Post graduate degree...स्नातकोतर स्तर पूरा ...... 06<br>No response......कोई उत्तर नहीं 88		
C 9	Which of the following best describes your main work status/ occupation over the past 12 months? पिछले 12 महीने में आपका <u>मुख्य</u>व्यवसाय क्या था है ? The purpose of this question is to assess their socio-economic status. If more than one,	Professional...प्रोफेशनल ....01<br>Medium or large Businessमध्य या बड़ा व्यवसाय .02<br>Middle / Senior Executive/officer in organization...मध्य या उच्च कार्यकारी 03<br>Agricultural land owner......जमींदार ....04<br>Sales and Marketing executives/ Clerical.... बिक्रीऔरविपणनकार्यकारी..या लिपिक ....05<br>Self-employed and small business ...स्व नियोजित या छोटा व्यवसाय ...06<br>Skilled manual laborer...कुशल श्रमिक ....11<br>Unskilled manual/agricultural laborer..अकुशल श्रमिक या कृषि श्रमिक ....12<br>Student.........छात्र .... 13<br>Homemaker.......गृहणी 14	| | | If fills 18 write down verbatim:<br>________<br>________<br>________<br>________<br>________	

	choose whichever was for the longer period in last 12 months. If not sure where the response would fit in please record verbatim and code it later after clarification.	Retired ....सेवा निवृत्त...............15<br>Unemployed (able to work) ...बेरोजगार...........16<br>Unemployed (unable to work) बेरोजगार (काम नहीं कर सकते)....17<br>No response ...कोई उत्तर नहीं........88<br>**If cannot ascribe to one of the above categories ..**18 **note down verbatim to code later**		

Section 1 .B BEHAVIOURAL MEASUREMENTS				
**Smoking/Tobacco use:**				
Now I am going to ask you some questions about various health related behaviors this will include questions on smoking, drinking alcohol, eating fruits and vegetables and physical activity. We have to ask these questions as written, so please do not take offense as none is intended.. Let's start with tobacco use.<br>अब मै आपसे कुछ अलग अलग स्वास्थ्य सम्बंधित व्यवहारों के बारे में प्रश्न पूछने लगा/ लगी हूँ। इन प्रश्नों में तम्बाकू सेवन , मदिरा सेवन , फल तथा सब्जियों के सेवन एवं शारीरिक गतिविधियों पर प्रश्न सम्मिलित होंगे। यह लाजमी है की हम हर लिखे गए प्रश्न को पूछें इस लिए कुपया पूछे जाने वाले प्रश्नों के कारण बुरा न मनाएं। शुरुआत तम्बाकू से सम्बंधित प्रश्नों से करते हैं				
**Questions and Filter**		**Response**		**Skip**
T1	Do you **currently** smoke any **tobacco** products, such as bidis, cigarettes, cigars, chilam, pipes, hookah or any other local smoked tobacco products? *(Use show card)*<br>क्या आप आजकल किसी भी प्रकार का धुम्रपान , जैसे कि बीड़ी ,सिगरेट,सिगार, चिलम, पाइप , हुक्का इत्यादि (शो कार्ड दिखाएँ) करते हैं ? Ask the participant to think of any	Yes ...हाँ .....1<br>No...नहीं 2	☐	*If No, go to T6*<br>*यदि नहीं तो T6 पर जाएँ*

Individual ID | | | | | | | |

	tobacco products he/she is smoking currently			
T2	Do you smoke tobacco products **daily**?<br>क्या आप रोजाना धूम्रपान करते हैं ?<br>This question is only for current smokers of tobacco products. Emphasize DAILY	Yes ...हाँ .....1<br>No...नहीं 2	☐	
T3	On an average, **how many** (number of times in case of hookah) of the following products do you smoke **each day/week**? आप औसतन एक दिन या एक हफ्ते में धुम्रपान के इन पदार्थों का कितना सेवन करते हैं (हुक्का के लिये कितनी बार)<br>➢ *Record for each type, Use Show Card*<br>➢ *Record 00, if any product is not used instead of leaving blank in the products categories.*<br>➢ *Record for any <u>new form of tobacco use</u> reported by the Respondent but interpret the answers carefully e.g.if someone reportsreverse smoking bidi it relates to a different method of smoking but the product remains bidi and should be marked as such.*<br>➢ *If less than daily, record weekly.* ***Fill only one of the two columns for each option****. The other column would be then marked 99*<br>➢ *If Don't Know 77*For current smokers only. Record dailyconsumption for daily smokers. If some products are smoked less than daily by daily smokers (for example a daily cigarette smoker may use bidi occasionally), enter weekly consumption. Also enter weekly consumption for current, non-daily smokers, remember that number of sticks or times(in case of Hokkah) need to be mentioned and not number of days			
		**<u>Daily Frequency</u>**<br>**<u>रोज की अव्वृति</u>**	**<u>Weekly Frequency</u>**<br>**<u>साप्ताहिक अव्वृति</u>**	
T3a	Bidis बीड़ी	☐☐	☐☐	
T3b	Manufactured Cigarettes सिगरेट	☐☐	☐☐	
T3c	Hand-rolled Cigarettes हाथ से बनी सिगरेट	☐☐	☐☐	

Individual ID

T3d	Pipes पाईप /Chilam चिलम			
T3e	Cigars, Cheroots सिगार , चेरूट			
T3f	Hookah/No. of Shisha session हुक्का या शीशा के सत्र			
T3g	E- cigarette इ सिगरेट			
T3h	Other local **smoked** tobacco products अन्य स्थानीय तम्बाकू के धुम्रपान के पदार्थ			
T3i	Other (Please Specify) अन्य (स्पष्ट करें)			
T4	During the past 12 months, have you tried **to stop smoking** क्या पिछले 12 महीने में आपने धुम्रपान छोड़ने की कोशिश की है?<br>For current smokers only. Ask the participant to think of any quit attempt they have made during the past 12 months. This has to be a real attempt and not just a thought.	Yes ...हाँ .....1<br>No...नहीं 2		
T5	During any visit to a doctor or other health worker in the past 12 months, were you advised to quit smokingtobacco?<br>क्या पिछले 12 महीने में किसी डॉक्टर या स्वास्थ्य कार्यकर्ता से परामर्श के दौरान उन्होंने आपको धुम्रपान छोड़ने की सलाह दी<br>For current smokers only. Ask the participant to think of visits to a doctor or other health worker during the past 12 months. If no visit, select "no visit during the past 12 months".	Yes ...हाँ .....1<br>No...नहीं ......2<br>No visit in the last 12 months period.<br>पिछले 12 महीनो में कोई परामर्श नहीं.................3		*If T2=2 go to T7*<br>*If T2=1 go to T8*
T6	In the **past**, did you **ever** smoke any tobacco products? (*Use show card)*<br>क्या आप पहले **<u>कभी भी</u>** धुम्रपान करते थे ?<br>Ask the participant to think of the time when he/she may have been smoking tobacco products.	Yes ...हाँ .....1<br>No...नहीं......2		*If No go to* T11<br>*अगर उत्तर=नही* T11 *पर जाएँ*

Individual ID

T7	In the past, did you **ever** smoke **daily**?<br>क्या आप पहले कभी भी **रोज़ाना** धुम्रपान करते थे ?<br>Ask the participant to think of the time when he/she may have been smoking tobacco products on a daily basis.	Yes ...हाँ .....1<br>No...नहीं ...2		*If No and T1=1 GO TO T11*<br><br>*If No and T1=2 go to T9*
T8	How old were you when you **first** started smoking**daily**? जब आपने पहली बार रोजाना धुम्रपान शुरू किया था तब आपकी उम्र कितनी थी<br>Ask the participant to think of the time when he/she started to smoke any tobacco products daily.	Age in completed years पूर्ण हुई उम्र<br>Don't remember याद नहीं ...77		*If T1=1 Go TO T11*
T9	How old were you when you **stopped** smoking?<br>जब आपने धुम्रपान करना बंद किया तब आपकी उम्र कितनी थी<br>Ask the participant to think of the time when he/she stopped smoking tobacco products	Age in completed years पूर्ण हुई उम्र<br>Don't remember याद नहीं...77		*If provided go T11 अगर उत्तर मिलता है तो T11 पर जाएँ*
T10	How **long ago** did you stop smoking?<br>आपने कितने साल पहले धुम्रपान करना बंद किया<br>If the participant doesn't remember his/her age when they stopped smoking, then record the time in years as appropriate	years साल<br>Don't remember याद नहीं...77		

Individual ID | | | | | | | |

**Smokeless Tobacco use**				
**Now I shall ask you about smokeless tobacco like** chewing tobacco, *tuibu* snuff, tobacco containing betel, gutka, , etc. **अब मै आपको धुम्रपान रहित तम्बाकू जैसे की चबाने वाला तम्बाकू , ताइबू, सनफ, पान के साथ तम्बाकू, गुटखा , इतियादी के सेवन से सम्बंधित प्रश्न पूछूँगा / पूछूँगी**				
**Questions**		**Response**		**Skip**
T11	Do you **currently use** any **smokeless tobacco**, such as (chewing tobacco, *tuibu* snuff, tobacco containing betel, gutka, pan masala, etc.)? क्या आप आजकल किसी भी प्रकार के धुम्रपान रहित तम्बाकू पदार्थ जैसे कि चबानेवालातम्बाकू, ताइबू, सनफ, पानकेसाथतम्बाकू, गुटखाइत्यादि(शो कार्ड दिखाएँ) का सेवन करते हैं ? Ask the participant to think of any smokeless tobacco products that he/she is using currently.	Yes …हाँ …..1<br>No…नहीं …2	☐	*If No, go to T14* *अगर उत्तर=नहीं T14 पर जाएँ*
T12	If yes, Do you **currently use smokeless tobacco** products **daily** क्या आप रोजाना धूम्रपान रहित तम्बाकू पदार्थ का सेवन करते हैं ? For current users of smokeless tobacco products only.	Yes …हाँ …..1<br>No…नहीं …2	☐	*If T12=2 go to T15*<br>*If T12=1 go to T16*
T13	On average, how many**times a day/week** do you use … आप औसतन एक दिन या एक हफ्ते में धुम्रपान रहित इन पदार्थों का कितना सेवन करते हैं<br>➢ *Record for each type, Use Show Card*<br>➢ *Record 00, if any product is not used instead of leaving blank in the products categories..*<br>➢ *If less than daily, record weekly. **Fill only one of the two columns for each option**. The other column would be then marked 99*<br>➢ *Don’t Know 77*<br>For current users of smokeless tobacco only. Record for each type of smokeless tobacco products, daily consumption for daily users. If products are used less than daily by daily users, For example a daily tobacco chewer			

Individual ID | | | | | | | |

	may use Betel leaves occasionally, then enter weekly consumption. Also enter weekly consumption for current, non-daily users.			
		Daily Frequency<br>रोज की अव्वृति	Weekly Frequency<br>साप्ताहिक अव्वृति	
T13a	Chewing tobaccoचबानेवालातम्बाकू			*If T12=2 go to T15 If T12=1 , go to T16*
T13b	Pan with zarda, Betel with Tobacco, quidपानकेसाथतम्बाकू/ज़र्दा			
T13c	Tuibu, Tobacco Snuff, by mouth<br>ताइबू,, सनफ (मुंह से)			
T13d	Snuff, by nose<br>सनफ (नाक से)			
T13e	Other (specify) अन्य (स्पष्ट करें)<br>………………………………..			
T14	If you are not using tobacco currently, In the **past** did you **ever use** smokeless tobacco products such as chewing tobacco, tuibu, snuff, betel, gutaka, etc.?<br>अगर आप अभी धुम्रपान रहित तम्बाकू पदार्थों जैसे कि चबानेवालातम्बाकू, ताइबू, सनफ, पानकेसाथतम्बाकू, गुटखा, पणमसालाया अन्य किसी पदार्थ का प्रयोग नहीं कर रहे तो क्या आप पहले कभी भी इनका प्रयोग करते थे ?<br>Ask the participant to think of the time when he/she may have been using smokeless tobacco products	Yes …हाँ …..1<br>No…नहीं …2		*If No go to T18*<br>*अगर उत्तरनहीं तो T18 पर जाएँ*

T15	In the **past**, did you **ever** use smokeless tobacco products such as [snuff, chewing tobacco, or betel] **daily**?<br>क्या आप पहले कभी भी धुम्रपान रहित तम्बाकू पदार्थों जैसे कि चबानेवालातम्बाकू, ताइबू, सनफ, पानकेसाथतम्बाकू, गुटखाया अन्य किसी पदार्थ का प्रयोग **रोज़ाना** करते थे ?<br><br>Ask the participant to think of the time when he/she may have been using smokeless tobacco products on a daily basis.	Yes …हाँ …..1<br>No…नहीं …2		*If No and T11=1 GO TO T18*<br><br>*If No and T11=2 go to T17*
T16	How old were you at that time when you **first started** using smokeless tobacco **daily**?<br>जब आपने सबसे पहले रोजाना धुम्रपान रहित तम्बाकू का सेवन शुरू किया तब आपकी उम्र क्या थी<br>Ask the participant to think of the time when he/she started to using any smokeless tobacco products.daily	Age in completed years<br>पूर्ण हुई उम्र<br>Don't remember याद नहीं…77		*If T11=1 Go TO T18*
T17	How old were you when you **stopped** taking smokeless tobacco products?<br>जब आपने धुम्रपान रहित तम्बाकू का सेवन बंद किया तब आपकी उम्र कितनी थी<br>Ask the participant to think of the time when he/she stopped using smokeless tobacco products.	Age in completed years<br>पूर्ण हुई उम्र<br>Don't remember याद नहीं…77		

	Can ask to correlate with special incidents in the person life or major well known events			

Passive Smoking				
Now I shall ask you about exposure to smoke because of smokers near you who share room/ space with you even if you are not smoking अब मै आपको धुम्रपान करने वाले व्यक्तियों के साथ कमरा या जगह साँझा करने के कारन होने वाले धुम्रपान के धुंए के एक्सपोजर (जब धुआं आप तक पहुंचा हो) के बारे में पूछूँगा / पूछूँगी ।				
T18	During the past 30 days, did someone smoke in your home (including any visitors) in your presence?<br>पिछले 30 दिनों में क्या किसी ने भी (जिनमे आगंतुक भी शामिल हैं) आपके घर में धुम्रपान किया जब आप वहां मौजूद थे ?<br>The participant should only think about other people, notabout him-/herself. Smokers should exclude themselves. The question is asking about inside the participant's home. This only includes fully enclosed areas of the home.	Yes …हाँ …..1<br>No…नहीं …2<br>Do not know.नहींजानते…7<br>No response/refuse…8		
T19	During the past 30 days, did someone smoke in closed areas in your workplace (in the building, in a work area or a specific office) in your presence?<br>क्या पिछले 30 दिनों में आपके कार्य स्थल में आपकी मौजूदगी में (बंद जगह जैसे कि कमरे में) किसी ने धुम्रपान किया ?<br>For those not working in a closed area, record "don't work in a closed area". Ask the participant to think of seeing somebody smoke or smelling the smoke in indoor areas at work during the past 30 days.	Yes …हाँ …..1<br>No…नहीं …2<br>Do not know…नहीं जानते 7<br>Do not work in closed areas बंद जगह में काम नहीं करते …8<br>Do not go out forwork कहीं काम ही नहीं करने जाते…9<br>No response/refuse…10		

Individual ID | | | | | | | |

T20	During the past 30 days, did someone smoke while you were travelling in car/ bus/ train/metro etc. ?<br>क्या पिछले 30 दिनों में कितने दिन कार या बस या ट्रेन या मेट्रो में सफर के दौरान आपकी मौजूदगी में किसी ने धुम्रपान किया?<br>Ask the participant to think of seeing somebody smoke or smelling the smoke in a vehicle while travelling during the past 30 days.	Yes ...हाँ .....1<br>No...नहीं ...2<br>Do not knowनहीं जानते..7<br>No response/refuse...8<br>Did not travel in a vehicle the last 30 days पिछले 30 दिन में किसी वाहन में सफर नहीं किया ...9	☐	

**Alcohol Consumption**				
The next questions relate to consumption of alcohol. As already said please do not get offended and answer truthfully as this information is confidential अब मैं आपसे शराब के सेवन से सम्बंधित प्रश्न पूछूँगा / पूछूँगी |जैसे आपको पहले बताया यह लाजमी है की हम हर लिखे गए प्रश्न को पूछें इस लिए कुपया पूछे जाने वाले प्रश्नों के कारण बुरा न मनाएं आपके द्वारा दी गयी जानकारी गोपनीय है इस लिए केवल सच बोलें |				
	**Questions**	**Responses**		**Skip**
A1	Have you **ever** consumed any alcoholic products (such as beer, wine, whisky, locally prepared alcohol, etc.)?<br>क्या आपने कभी भी किसी भी प्रकार की शराब (जैसे कि बीयर, रम , व्हीस्की ब्रैंडीदेसी, ताड़ी इतियादी) का सेवन किया है ?<br>➢ *Use Showcard /show examples*<br>Ask the participant to think of any drink that contains alcohol, with the exception of alcohol-based medication taken due to health reasons or alcohol consumed for religious reasons.	Yes ...हाँ .....1<br>No...नहीं ...2	☐	*If No, go to D1*<br>*अगर उत्तरनहीं तो D1 पर जाएँ*
A2	How **old** were you when you **first** consumed alcohol?<br>जब आपने सबसे पहले शराब का सेवन किया तब आपकी उम्र कितनी थी ?<br>Ask the participant to think of the age when he/she started consuming alcohol	Age in completed years<br>पूर्ण हुई उम्र<br>Don't remember याद नहीं...77	☐☐	
A3	Have you consumed any alcoholic products within the **past 12months**?	Yes ...हाँ .....1No...नहीं ...2	☐	*If No, go to*

	क्या आपने पिछले 12 महीनो में शराब का सेवन किया है ? Ask the participant to think of any drinks that contain alcohol,with the exception of alcohol-based medication that is taken dueto health reasons or for religious reasons in the last 12 months			A15 अगर उत्तरनहीं तो A15 पर जाएँ
A4	During the past 12 months, how often have you found that you were **not able to stop** drinking once you had started? पिछले 12 महीने में आपने ऐसा कितनी बार पाया कि एक बार पीना शुरू करने के बाद आप अपने को रोक नहीं पायें हो अथवा तब तक पिया हो जब तक होश नहीं गवां दी हो Ask the participant to think of the past 12 months. Read out all the answer options .	Daily or almost daily रोज़ाना.या लगभग रोज़ाना..1<br>Weekly हर हफ्ते .2<br>Monthlyहर महीने ...3<br>Less than monthlyहर महीने से कम ....4<br>Never.कभी नहीं . ...5<br>Does not know ...नहीं जानते ..7<br>No response कोई जवाब नहीं दिया 8	☐	
A5	During the past 12 months, how often have you **failedto do** what was **normally expected** from you because of drinking? पिछले 12 महीने में आपने ऐसा कितनी बार पाया कि पीने के कारण आप वह करने में असक्षम रहें हों जो आपसे अपेक्षित था Ask the participant to think of the past 12 months. Read out all the answer options	Daily or almost daily रोज़ाना.या लगभग रोज़ाना..1<br>Weekly हर हफ्ते .2<br>Monthly हर महीने ...3<br>Less than monthly हर महीने से कम ....4<br>Never.कभी नहीं . ...5<br>Does not know ...नहीं जानते ..7<br>No response कोई जवाब नहीं दिया 8	☐	
A6	During the past 12 months, how often have you **neededa first drink** in the morning to get yourself going after a heavy drinking session? पिछले 12 महीने में आपने ऐसा कितनी बार पाया कि बीती रात को ज्यादा शराब पीने के बाद	Daily or almost daily रोज़ाना.या लगभग रोज़ाना.......1<br>Weekly हर हफ्ते .2<br>Monthly हर महीने ...3<br>Less than monthly हर महीने से कम ....4<br>Never.कभी नहीं 5	☐	

Individual ID | | | | | | | |

	सुबह उठते ही आपको शराब पीने की जरुर पड़ी हो ?<br><br>Ask the participant to think of the past 12 months. Read out all the answer options.	Does not know ...नहीं जानते ..7<br>No response कोई जवाब नहीं दिया 8		
Spend some time trying to understand the alcohol use pattern of the respondent and estimate most appropriate standard drink size				
A7	In past 12 months, **how frequently** have you had at least one standard alcoholic drink (clarify one standard drink to the respondent using the calculations done above)? पिछले 12 महीने में कम से कम एक स्टैण्डर्ड ड्रिंक का (जिसको मै शो कार्ड द्वारा आपको समझा रहा हूँ)आपने कितनी आव्वृति से सेवन किया होगा<br>➢ *Read Responses*<br>For those that have consumed alcohol in the past 12 months. A “standard drink” is the amount of ethanol contained in standard glasses of beer, wine, fortified wine such as sherry, and spirits. *Use show card.*	Daily रोज़ाना..1<br>5 - 6 days per week हफ्ते में 5 से 6 दिन.... 2<br>3 - 4 days per week हफ्ते में 3 से 4 दिन....3<br>1 -2 days per week हफ्ते में 1 से 2 दिन....4<br>1 - 4 days per month महीने में1 से 4दिन..5<br>Less than once a month महीने में 1 दिन सेकम..6	☐	
A8	Have you consumed any alcohol drink within the past **30 days**? पिछले30 दिनों में क्या आपने शराब का सेवन किया ?<br>Select the appropriate response	Yes ...हाँ .....1<br>No...नहीं ...2	☐	*If No, go toD1* *अगर उत्तरन हीं है तो D1पर जाएँ*
A9	During the past 30 days, on how many **occasions** did you have at least	Number नम्बर<br>Don't know ...नहीं जानते ...77	☐☐	

	one standard alcoholic drink? पिछले 30 दिनों में कितने अवसरों पर आपने कम से कम एक स्टैण्डर्ड ड्रिंक का सेवन किया ?<br><br>Ask the participant to think of the past 30 days only. Record the number of occasions. Note that there can be more than one occasion in which alcohol is consumed in a given day.			
A1<br>0	During the past 30 days, when you drank alcohol, how many **standard drinks on average** did you have during one drinking occasion? पिछले 30 दिनों में जब भी आपने शराब का सेवन किया , ऐसे एक अवसर पर आपने औसतन कितने स्टैण्डर्ड ड्रिंक का सेवन किया होगा ?<br>➢ *Use show card*<br>Help the participant to average out the total number of drinks by using the show card that shows standard alcoholic drinks	Number नम्बर<br>Don't know...नहीं जानते .. 77	☐☐	
A1<br>1	During the past 30 days, what was the **largest number** of standard drinks you had on a single occasion, counting all types of alcoholic drinks together? पिछले 30 दिनों में किसी भी एक पीने के अवसर पर आपके द्वारा सर्वाधिक स्टेंडर्ड ड्रिंक की संख्या क्या थी (उस दिन पी गयी सब तरह की शराब को जोड़ कर बताएं)?<br>Ask the participant to think of the past 30 days only. This question is about the largest number of drinks that the participant had on one single occasion.	Largest number सबसे ज्यादा नम्बर<br>Don't know...नहीं जानते .. 77	☐☐	
A1<br>2	During the past 30 days, how many times did you have Six **or more Drinks** पिछले 30 दिन में कितनी बार आपने एक दिन में 6 या उस से ज्यादा स्टेंडर्ड ड्रिंक्स का सेवन किया?	Number of times नम्बर<br>Don't know...नहीं जानते .. 77	☐☐	

	Ask the participant to think of the past 30 days only, and to report the number of occasions when he/she had six or more standard drinks. Note that the question is NOT different for men and women.			

A13a	During each of the past 7 days, how many standard drinks of any alcoholic drink did you have each day? बीते 7 दिनों में प्रत्येक दिन आपने किसी भी प्रकार की शराब की कितनी स्टैण्डर्ड ड्रिंक्स का सेवन किया ?	Monday सोमवार		
A13b		Tuesday मंगलवार		
A13c		Wednesday बुधवार		
A13d		Thursday वीरवर		
A13e	➢ *Record for each day*	Friday शुक्वर्वार		
A13f	➢ *Use Show Card for Standard Drink*	Saturdayशनिवार		
A13g	➢ *Don't know 77*<br>Ask the participant to think of each of the past 7 days. Use the showcard that shows standard alcoholic drinks to help the participant report the number of standard drinks for each of the past 7 days. Record for each day the number of standard drinks. If no drinks record 0. Start from the previous day and go backwards.	Sunday रविवार		

I have just asked you about your consumption of alcohol during the past 7 days. The questions were about alcohol in general, while the next questions refer to your consumption alcohol from source other than authorized shop like smuggled in alcohol or, home brewed alcohol. Please think only about these types of alcohol when answering the next questions. मैंने अभी आपसे पिछले 7 दिन में शराब के सेवन के बारे में बात की यह शराब के बारे में कुछ सामान्य सवाल थे , अब मै आपसे अधिकृत शराब के स्रोत्रों के आलावा प्राप्त की गयी शराब (जैसे की तस्करी से या कच्ची शराब जो घर पर या भट्टी पर बनी हो)के सेवन से सम्बंधित सवाल पूछूँगापूछूँगा / पूछूँगी इसलिए अगले कुछ प्रश्नों के लिए केवल इस प्रकार की शराब के बारे में सोच कर उत्तर दें

Individual ID

A 14	During the past 7 days, did you consume alcohol from source other than authorized shop? क्या पिछले 7 दिन में आपने अधिकृत स्रोत्रों के आलावा किसी स्रोत्र से ले कर शराब का सेवन किया ?<br>➢ *Use show card*<br>Ask the participant to only think of homebrewed alcohol or alcohol brought across national border.	Yes ...हाँ .....1No...नहीं ...2		*If No, go toD1अगर उत्तरनहीं है तो D1पर जाएँ*
A14a	If yes, Did you consume alcohol smuggled in (therefore untaxed) from another country (not another state)? क्या आपने विदेश से तस्करी कर लायी गयी(जिस पर टैक्स नहीं लगा हो)शराब का सेवन किया ?	Yes ...हाँ .....1<br>No...नहीं ...2		
A14b	If yes, on average, how many standard drinks of the alcohol smuggled in (therefore untaxed) from another country (not another state) did you consume during the past 7 days? पिछले 7 दिन में औसतन आपने तस्करी कर के दूसरे देश से लायी गयी शराबके कितने स्टैण्डर्ड ड्रिंक पीये<br>➢ *Don't Know 77*			
A14c	During the past 7 days Did you consume home brewed alcohol? क्या पिछले 7 दिन में आपने घर पर बनी कच्ची शराब का सेवन किया ?	Yes ...हाँ .....1<br>No...नहीं ...2		
A 14d	If yes, on average, how many standard drinks of the home brewed alcohol did you consume during the past 7 days? पिछले 7 दिन में औसतन आपने घर पर बनी कच्ची शराब के कितने स्टैण्डर्ड ड्रिंक पीये<br>➢ *Don't Know 77*			
A14e	During the past 7 days did you consume illegally brewed alcohol? क्या पिछले 7 दिन में आपने भट्टी पर बनी कच्ची शराब का सेवन किया ?	Yes ...हाँ .....1<br>No...नहीं ...2		

Individual ID

A14 f	If yes, on average, how many standard drinks of the illegally brewed alcohol did you consume during the past 7 days? पिछले 7 दिन में औसतन आपने भट्टी पर बनी कच्ची शराब के कितने स्टैण्डर्ड ड्रिंक पीये<br>➢ *Don't Know 77*			
A14g	During the past 7 days did you consume alcohol not intended for drinking, e.g. alcohol-based medicines, perfumes, after shaves ? क्या पिछले 7 दिन में आपने शराब जो की पीने के लिया नहीं बल्कि ओधोगिक इस्तेमाल के लिए बनी हो का सेवन किया ?	Yes …हाँ …..1<br>No…नहीं …2		
A14h	If yes on average, how many standard drinks of the alcohol not intended for drinking, e.g. alcohol-based medicines, perfumes, after shaves etc…did you consume during the past 7 days? पिछले 7 दिन में औसतन आपने शराब जो की पीने के लिया नहीं बल्कि ओधोगिक इस्तेमाल के लिए बनी हो के कितने स्टैण्डर्ड ड्रिंक पीये<br>➢ *Don't Know 77*			
A14i	During the past 7 days did you consume any other untaxed alcohol in the country क्या पिछले 7 दिन में आपने अन्य कोई शराब जिस पर टैक्स नही लगा हो का सेवन किया ?	Yes …हाँ …..1<br>No…नहीं …2		
A 14 j	If yes, on average, how many standard drinks of any other untaxed alcohol in the country did you consume during the past 7 days? पिछले 7 दिन में औसतन आपने अन्य कोई शराब जिस पर टैक्स नही लगा हो के कितने स्टैण्डर्ड ड्रिंक पीये<br>➢ *Don't Know 77*			

Individual ID | | | | | | | |

A15	Have you stopped drinking due to health reasons, such as a negative impact on your health or on the advice of your doctor or other health worker?क्या आपने स्वास्थ्य कारणों से जैसे कि आपके स्वास्थ्य पर पड़े बुरे प्रभाव या फिर किसी डॉक्टर या स्वास्थ्य कर्मी की सलाह पर शराब पीना छोड़ दिया है ?	Yes…हाँ….1<br>No…नहीं …2		

Diet				
The next questions I will ask about the fruits and vegetables that you usually eat. I have a nutrition card here that shows you some examples of local fruits and vegetables. Each picture represents the size of a serving. As you answer these questions please do not think only of the past one week (which might be different from what you usually do - like there might have been a family occasion last week or you could have been out vacationing last week) instead think of a '**typical' or a 'usual**' week. Look at daily consumption as a whole by clubbing together various dishes/ sessions of eating<br>अब मै आपसे आपके सामान्यत: किये जाने वाले फल तथा सब्जियों के सेवन के बारे में पूछूँगा / पूछूँगी | इस कार्ड पर कुछ स्थानीय फल तथा सब्जियों के चित्र हैं हर एक के आगे एक परोसन की अंदाज़न मात्रा दर्शायी गयी है | यह जरुरी है कि मेरे प्रश्नों का उत्तर देते समय आप अपने एक सामान्य हफ्ते के बारे में सोचें न की केवल पिछले एक सप्ताह के बारे में (क्योंकि पिछला हफ्ता आपके सामान्य हफ्तों से अलग भी हो सकता है जैसे की कोई पारिवारिक समारोह रहा हो सकता है या आप घूमने के लिए कहीं बाहर गए हो सकते हैं जिस की वजह से आपके सामान्य खानपान में बदलाव आया हो सकता है). अपने रोज़ के फल अथवा सब्जी खाने के बारे में सोचें इसमे दिन भर में अलग अलग समय पर खाए गए अलग-अलग पकवानों के फल एवं सब्जियों की मात्रा को ध्यान में रक् कर उत्तर दें				
**Questions**		**Response**		**Skip**
D1	In a typical week, on how many days do you **eat fruit**? एक सामान्य हफ्ते में आप कितने दिन फल खाते हो ?<br>*(Use show card)*<br>Ask the participant to think of any fruit on the show card. A typical week means a "normal" week when the diet is not affected by cultural, religious, or other events. Ask the participant to not report an average over a period.	Number of days दिनों की संख्या<br>Don't Know नहीं जानते …77		*If zero days go to D3*<br>*अगर शून्य दिन तो D3 पर जाएँ*

D2	How many **servings** of fruit do you eat on **one** of those days? जिन दिनों आप फल खाते हो , आप सामान्यत: फल की कितनी परोसन लेते हो ?<br>*(Use show card)*<br>Ask the participant to think of one day he/she can recall easily. Refer to the show card for serving sizes. Assist them in estimating right serving size.	Number of servings परोसन की संख्या<br>Don't Know… नहीं जानते 77		
D3	In a typical week, how many days do you **eat vegetables**? एक सामान्य हफ्ते में आप कितने दिन सब्जियां खाते हो ?<br>*(Use show card)*<br>Ask the participant to think of any vegetable on the showcard. A typical week means a "normal" week when the diet is not affected by cultural, religious, or other events. Ask the participant to not report an average over a period.	Number of days दिनों की संख्या<br>Don't Know नहीं जानते …77		*If zero days go to D5*<br>*अगर शून्य दिन तो D5पर जाएँ*
D4	How many **servings** of vegetables do you eat on one of those days? जिन दिनों आप सब्जियां खाते हो , आप सामान्यत: सब्जियों की कितनी परोसन लेते हो ?<br>*(Use show card)*<br>Ask the participant to think of one day he/she can recall easily. Refer to the showcard for serving sizes. Assist them in estimating right serving size.	Number of servings परोसन की संख्या<br>Don't Know… नहीं जानते 77		
D5	In a typical week, on how many days do you **drink fruit or vegetable juice (this includes fresh juice made at home/shop or, packed juice- but does not include flavored drinks**? एक सामान्य हफ्ते में आप कितने दिन फल अथवा सब्जियों का जूस पीते हो ? (इस में घर में बना , दूकान में बना अथवा टिन बंद जूस शामिल है परन्तु फ्लेवर्ड पेय शामिल नहीं हैं)<br>*(Use show card)*<br>Ask the participant to think of any fruit on the showcard. A typical week means a	Number of days दिनों की संख्या<br>Don't Know नहीं जानते …77		*If zero days go to D7*<br>*अगर शून्य दिन तो D7पर जाएँ*

	"normal" week when the diet is not affected by cultural, religious, or other events. Ask the participant to not report an average over a period.			
D6	How many **servings** of fruit or vegetable juice do you consume on one of those days that you drink juice? जिन दिनों आप फल अथवा सब्जियों का जूस पीते हो , आप सामान्यत: जूस की कितनी परोसन लेते हो ?<br>*(Use show card)*<br>Ask the participant to think of one day he/she can recall easily. Refer to the showcard for serving sizes. Assist them in estimating right serving size.	Number of servings परोसन की संख्या<br>Don't Know... नहीं जानते 77	☐☐	

**Dietary Salt**
With the next questions, we would like to learn more about salt in your diet. Dietary salt includes ordinary table salt<br>अगले कुछ प्रश्न आपके खाने में नमक के बारे में हैं. खाने के नमक में उपर से डाले जाने वाला साधारण नमक भी शामिल है<br>➢ *Show card*<br>The following questions pertain to adding salt to the food right before you eat it, food preparation at your home and on controlling your salt intake. Please answer the questions even if you consider yourself taking a diet low in salt. अगले कुछ प्रश्न खाने से ठीक पहले खाने में नमक डालने से, घर में खाना बनाने से एवं अपने खाने के नमक की मात्रा को नियंत्रित करने से सम्बंधित है I<br>Read this opening statement out loud. Don't forget to use the showcard which will help the respondent when answering to the questions

D7	How often do you add **extra salt** to your food right before you eat it or as you are eating it? आप कितनी बार खाना खाते समय खाने में ऊपर से अतिरिक्त नमक डालते हैं ?<br>(*Select One only)*<br>Read out all the answer options.	Never...कभी नहीं ..0<br>Always...हमेशा .. 1<br>Often...कई बार .. 2<br>Sometimes...कभी कभी .. 3<br>Rarely कदाचित ...4<br>Don't know नहीं जानते..... 7	☐	

Individual ID | | | | | | | |

D8	Now tell me how often do you take each of the following **food having high salt content including processed/ prepacked ready to eat food items?** अब आप मुझे बताएं कि आप इन अधिक नमक वाले खाद्य पदार्थों का सेवन कितनी आवृत्ति से करते हैं ?		
D8a	Namkeen, papad, packaged chips, soy or mustard sauce etc<br>नमकीन पापड़ पैक्ड चिप्स सोय या मस्टर्ड सौस इत्यादि<br>➢ *Use show card*<br>*Read out each option*	Never...कभी नहीं ..0<br>Daily...रोजाना ..1<br>5-6 days per weekहफ्ते में 5 से 6 दिन..2<br>3-4 days per week हफ्ते में 3 से 4 दिन..3<br>1-2 days per week. हफ्ते में 1 से 2 दिन..4<br>1-3 days per month महीने में 1 से 3 दिन..5<br>Less than once a month महीने में एक से कम दिन..6<br>Don't know ..नहीं जानते...7	
D8b	Preserved canned salty products including pickle<br>संरक्षित (प्रेसेर्वेड) नमकीन डिब्बा बंद पदार्थ जैसे डिब्बा बंद आचार<br>➢ *Use show card*<br>*Read out each option.*<br>*Remind the participant that this is only for preserved food.*	Never...कभी नहीं ..0<br>Daily...रोजाना..1<br>5-6 days per weekहफ्ते में 5 से 6 दिन 2<br>3-4 days per week हफ्ते में 3 से 4 दिन..3<br>1-2 days per week. हफ्ते में 1 से 2 दिन..4<br>1-3 days per month महीने में 1 से 3 दिन....5<br>Less than once a month महीने में एक से कम दिन 6<br>Don't know ..नहीं जानते .7	
D8c	Homemade high salt content food like chutney, panna, pickle.<br>घर में बनी ज्यादा नामक वाली चटनी , पन्ना , आचार इतियादी<br>➢ *Use show card*<br>*Read out each option*<br>*Remind the participant that this is only for homemade food.*	Never...कभी नहीं .0<br>Daily...रोजाना 1<br>5-6 days per weekहफ्ते में 5 से 6 दिन 2<br>3-4 days per week हफ्ते में 3 से 4 दिन..3<br>1-2 days per week. हफ्ते में 1 से 2 दिन..4<br>1-3 days per month महीने में 1 से 3 दिन....5<br>Less than once a monthमहीने में एकसे कम दिन..6<br>Don't know ..नहीं जानते .7	
D8d	Other dairy products having salt like processed or packaged cheese, butter etc. This is different from home made butter or cheese - this pertains to processed products in	Never...कभी नहीं .0<br>Daily...रोजाना 1<br>5-6 days per weekहफ्ते में 5 से 6 दिन 2<br>3-4 days per week हफ्ते में 3 से 4 दिन..3<br>1-2 days per week. हफ्ते में 1 से 2 दिन..4<br>1-3 days per month महीने में 1 से 3 दिन....5	

	which salt is added as preservative or for taste) अन्य दुग्ध उत्पाद जिन में नमक होता है जैसे परिष्कृत (प्रोसेस्ड) चीज़ (डब्बा बंद पनीर) , मक्खन इतियादी (यह घर में निकले मक्खन या पनीर से अलग है - यह परिष्कृत पदार्थों से सम्बंधित है जिस में नमक को परिरक्षक की तरह या स्वाद बदलने के लिए प्रयोग में लाया जाता है)<br>➢ *Use show card*<br>*Read out each option*<br>*Remind the participant that this is only for those that contain salt.*	Less than once a month महीने में एक से कम दिन 6<br>Don't know ..नहीं जानते .7	
D9	**How much salt or high salt containing products** do you think you consume? आप क्या सोचते हैं कि आप कितनी मात्रा में नमक या नमकीन पदार्थों का सेवन करते हैं?<br>This tests the participant's opinion on his/her salt consumption. There is no right answer.	Far too much कहीं ज्यादा मात्रा में करते हैं..1<br>Too muchज्यादामात्रामेंकरतेहैं .....2<br>Just the right amount बिलकुल सही मात्रा में करते हैं..3<br>Too little कम मात्रा में करते हैं .....4<br>Far too little बहुत कम मात्रा में करते हैं .....5<br>Don't know ...नहीं जानते 7	☐
D10	Do you think your daily salt intake **affects your health.** क्या आपको लगता है कि आपके द्वारा खाये नमक की मात्रा आपके स्वास्थ्य पर असर करती है ? Just take the participant opinion, do not ask leading question	Yes ...हाँ .....1<br>No...नहीं ...2<br>Don't know .. पता नहीं ..7	☐
D11	How important to you is **lowering of salt** in your diet? आपके लिए अपने खाने में नमक की मात्रा कम करना कितना महत्त्व रखता है<br>Just take the participant opinion, do not ask leading question	Very important बहुत महत्त्व रखता है ....1<br>Somewhat important कुछ महत्त्व रखता है..2<br>Not at all important बिलकुल महत्त्व नहीं रखता है.. 3<br>Don't know नहीं जानते ...7	☐

D12	Do you practice any of the following on a regular basis to control your salt intake? (Record for each) आप अपने नमक खाने पर नियंत्रण रखने के लिए इनमें से किन उपाय पर नियमित रूप से अमल करते हैं (हर उपाय के लिए निशान लगायें) Select the appropriate response for each option. Ask the participant to only consider actions that he/she undertakes specifically to control salt intake, and not for any other purpose

Individual ID | | | | | | |

D12 a	Limit consumption of high salt containing foods like papad ज्यादा नमक वाली चीज़े जैसे की पापड़ के खाने पर नियंत्रण	Yes …हाँ ..1<br>No…नहीं …2	☐	
D12 b	Look at the salt or sodium content on food labels खाद्य पदार्थों पर लगे लेबल पर नमक की मात्र को जांचना	Yes …हाँ ..1<br>No…नहीं …2	☐	
D12 c	Buy low salt/sodium alternatives(but mention this only if you use alternatives to reduce the sodium content and not if you take these as a habit or for taste) कम नमक/ कम सोडियम वाले विकल्प खरीद कर प्रयोग करना (अगर आप इस तरह का नमक केवल कम सोडियम की वजह से लेते हैं तभी बताएं - अगर आप यह नमक आदत या स्वाद के कारण लेते है तब नहीं)	Yes …हाँ ..1<br>No…नहीं …2	☐	
D12 d	Use spices other than salt when cooking to make up for taste<br>खाना पकाने के समय नमक के आलावा स्वाद ठीक करने के लिए अन्य मसाले प्रयोग में लाते हैं	Yes …हाँ ..1<br>No…नहीं …2	☐	
D12 e	Avoid eating foods prepared outside of a home घर में बने खाने के आलावा बाहर का खाना खाने से कतराते हैं	Yes …हाँ ..1<br>No…नहीं …2	☐	
D12 f	Do other things specifically to control your salt intake like not adding salt on table or not adding salt in flour नमक का सेवन कम करने के लिए अन्य कुछ करते हैंजैसे कि खाने में उपर से नमक नहीं डालते या आटे में नमक नहीं डालते (स्पष्ट करें)	Yes …हाँ ..1<br>No…नहीं …2	☐<br>Other (please specify)……………….	
D13	On average, in a **typical** week how many meals (by meals I mean breakfast, lunch or, dinner) do you eat **that were NOT home-cooked** (Home cooked means cooked at your own / relatives' or friends' home)? औसतन एक सामान्य हफ्ते में आप ऐसे कितने भोजन (ब्रेक फ़ास्ट / लंच या डिनर) खाते हैं जो कि घर पर नहीं पके होते (इस सन्दर्भ में घर का माने है आप का अपना घर या आपके रिश्तेदारों या दोस्तों का घर) Ask the participant to think of meals that were NOT prepared at home,(Home includes his/her own home, the home of other family members or friends)	Number of times नम्बर<br>Don't know…नहीं जानते .. 77	☐☐	

Physical Activity

Individual ID | | | | | | | |

Next I am going to ask you about the time you spend doing different types of physical activity in a typical week. Please answer these questions even if you do not consider yourself to be a physically active person. अब मै आपसे एक सामान्य सप्ताह में आपकी शारीरिक क्रिया शीलता के बारे में पूछने लगा हूँ | अगर आप अपने को शारीरिक रूप से क्रियाशील नहीं मानते तो भी इन प्रश्नों का उत्तर जरुर दें

Think first about the time you spend doing work. Work includes things that you have to do such as paid or unpaid work, study/training, household chores, harvesting food/crops, fishing or seeking food or fuel like wood, seeking employment. सर्वप्रथम उस समय के बारे में सोचें जिस में आप कार्यशील होते हैं | कार्य में यह सब आता है : कार्य जिसके लिए आपको भुगतान मिलता है या नहीं मिलता है, पढाई, किसी प्रकार की ट्रेनिंग, घर के काम-काज, फसल की कटाई, मछली पकड़ना , खाना या खानां पकाने की लिए इंधन जैसे लकडियों का इंतजाम करना या नौकरी की तलाश करना

In answering the following questions 'Vigorous -Intensity activities' are activities that require hard physical effort and cause large increase in breathing or heart rate, 'Moderate-Intensity activities' are activities that require effort and cause small increases in breathing or heart rate. अगले कुछ प्रश्नों के उत्तर देते समय यह ध्यान रखें कि भारी मेहनत शारीरिक क्रिया का अर्थ है वह क्रियाएं जिसमे शरीर को ज़ोर लगाना पड़ता हैं और जिस से श्वास लेने तथा ह्रदय गति काफी बढ़ जाती है | मध्यम मेहनत शारीरिक क्रिया का अर्थ है वह क्रियाएं जिसमे शरीर को कुछ कम ज़ोर लगाना पड़ता हैं और जिस से श्वास लेने तथा ह्रदय गति थोड़ी ही बढती है

Read this opening statement out loud. It should not be omitted. The respondent will have to think first about the time he/she spends doing work (paid or unpaid work, household chores, harvesting food, fishing or seeking food or fuel like wood, seeking employment [Insert other examples if needed]), then about the time he/she travels from place to place, and finally about the time spent in vigorous as well as moderate physical activity during leisure time.

Remind the respondent when he/she answers the following questions that 'vigorous-intensity activities' are activities that require hard physical effort and cause large increases in breathing or heart rate, 'moderate-intensity activities' are activities that require moderate physical effort and cause small increases in breathing or heart rate. Don't forget to use the show card which will help the respondent when answering to the questions

Use the information provided so far e.g. occupation and ask the respondent about his/ her routine for a typical day and try to understand the same it will help fill this section.

Individual ID | | | | | | | |

Questions		Response		Skip
P1	Does your routine work at home/ workplace involve **vigorous-intensity activity** that causes large increases in breathing or heart rate like (carrying or lifting heavy loads, digging or construction work) for **at least 10 minutes** continuously?क्या आपको अपने रोजमर्रा के काम में भारी मेहनत वाली शारीरिक क्रिया (जिस से श्वास लेने तथा ह्रदय गति काफी बढ़ जाती है) करनी पड़ती है जो किआप कम से कम 10 मिनट तक लगातार करते हो (जैसे भार उठाना, भारी सामान लेकर जाना, या खुदाई या निर्माण कार्य)<br>➢ *Use show card*<br>Ask the participant to think about vigorous-intensity activities at work only. Activities are regarded as vigorous intensity if they cause large increases in breathing and/or heart rate	Yes हाँ .1<br>No.नहीं...2	☐	*If No, go toP4*<br>*अगर उत्तरनहीं है तो P4पर जाएँ*
P2	In a typical week, on how many days do you do **vigorous-intensity activities** as part of your work at home/ workplace?<br>एक सामान्य हफ्ते में आप अपने घर या काम के दौरान कितने दिन भारी मेहनत वाली शारीरिक क्रियाकरते हैं ?<br>"Typical week" means a week when the participant is engaged in his/her usual activities. Valid responses range from 1-7.	Number of days<br>दिनों की संख्या	☐	
P3	On a typical day that you do **vigorous-intensity activities** how much time do you spend doing **vigorous-intensity** activity at home/work place?<br>एक सामान्यदिन जब आप घर या काम के दौरान भारी मेहनत वाली शारीरिक क्रिया करते हैं , तो उस में कितना समय बिताते हैं ?<br>Ask the participant to think of a typical day he/she can recall easily in which he/she engaged in vigorous-intensity activities at work. The participant should only consider those activities undertaken continuously for 10 minutes or more.	Hours<br>घंटे<br>Minutes<br>मिनट	☐☐<br>☐☐	

	Probe very high responses (over 4 hrs) to verify.			
P4	Does your work at home / workplace involve **moderate-intensity** activity that causes small increases in breathing or heart rate for at least 10 minutes continuously such as brisk walking or carrying loads, manual washing of clothes, dry sweeping of floor, wet mopping of floor, drawing water from well, carrying water from tap, carrying water from river or well, manual grinding or pounding of cereals, gardening at home etc. ?<br>क्या आपको अपने रोजमर्रा के काम में मध्यम मेहनत वाली शारीरिक क्रिया करनी पड़ती है जिस से श्वास लेने तथा ह्रदय गति कुछ बढ़ जाती है जो कि कम से कम 10 मिनट तक लगातार करते हो (जैसे तेज चलना , कुछ सामान उठा कर लाना ले जाना हाथ से कपडे धोना ,घर में झाड़ू या पोछा लगाना, कुँए से पानी निकालना, पीने का पानी स्रोत्र से भर कर लाना, अनाज कूटना, घर में बागवानी करना इत्यादि)<br><br>Ask the participant to think about moderate-intensity activities at work only. Activities are regarded as moderate intensity if they cause small increases in breathing and/or heart rate.	Yes हाँ .1<br>No.नहीं...2<br>No response..8	☐	*If No, Go to P7*<br>*अगर उत्तरनहीं है तो P7पर जाएँ*
P5	In a typical week, on how many days do you do **moderate-intensity** activities as part of your work at home/ workplace for **at least 10 minutes** continuously?<br>एक सामान्य हफ्ते में आप अपने काम के दौरान कितने दिन मध्यम मेहनत वाली शारीरिक क्रिया करते हैं ?<br>➢ *Use show card*<br>"Typical week" means a week when the participant is engaged in his/her usual activities. Valid responses range from 1-7.	Number of days<br>दिनों की संख्या	☐	

P6	On a typical day that you do **moderate-intensity activities** how much time do you spend doing moderate-intensity activity at home/work place?<br>एक सामान्यत: दिन जब आप घर या काम के दौरान माध्यम मेहनत वाली शारीरिक क्रिया करते हैं , तो उस में कितना समय बिताते हैं ?<br>Ask the participant to think of a typical day he/she can recall easily in which he/she engaged in moderate-intensity activities at work. The participant should only consider those activities undertaken continuously for 10 minutes or more. Probe very high responses (over 4 hrs) to verify.	Hours<br>घंटे<br>Minutes<br>मिनट		
While answering the next question please exclude any activities that you have already mentioned in the questions above on intensive or moderate physical activity. Now I will ask you about the usual way that you travel to and from places. For example to work, for shopping, to market, to place of worship , to fields etc<br>अगले प्रश्नों का जवाब देते समय उन सब गतिविधियों को हटा दें जो आप उपर पूछे भारी या मध्यम मेहनत वाली गतिविधियों केसवालों के जवाब में बता चुके हैं । अब मै आपसे कहीं भी आने जाने के आपके द्वारा इस्तेमाल किये जाने के सामान्य तरीके के बारे में पूछूँगा / पूछूँगी इसमे काम पर जाना, खेतों तक जाना पूजा के स्थान मंदिर या बाजार जाना इतियादी शामिल हैं ।				
P7	Do you walk or use a bicycle (pedal cycle) for at least 10 minutes continuously to get to and from places? क्या आप लगातार कम से कम 10 मिनट तक साइकिल चला कर या पैदल चल कर कहीं आते जाते हैं ?<br>Select the appropriate response.	Yes हाँ<br>.1No.नहीं...2		*If No, go to P10*<br>*अगर उत्तरनहीं है तो P10 पर जाएँ*
P8	In a typical week, on how many days do you walk or bicycle for at least 10 minutes continuously to get to and from places?<br>एक सामान्य हफ्ते में आप कितने दिन लगातार कम से कम 10 मिनट तक साइकिल चला कर या पैदल चल कर कहीं आते जाते हैं ?<br>“Typical week” means a week when the participant is engaged in his/her usual activities. Valid responses range from 1-7.	Number of days<br>दिनों की संख्या		
P9	On a typical day that you do **walk or bicycle** how much time do you spend walking or bicycling for travel?	Hours<br>घंटे<br>Minutes<br>मिनट		

	एक सामान्य दिन जब आप साइकिल चला कर या पैदल चल कर कहीं आते जाते हैं, तो उस में कितना समय बिताते हैं ?<br>Ask the participant to think of a typical day he/she can recall easily in which he/she engaged in transport-related activities. The participant should only consider those activities undertaken continuously for 10 minutes or more. Probe very high responses (over 4 hrs) to verify			

While answering the next question please exclude any activities that you have already mentioned in the questions on intensive or moderate physical activity or while travelling from or to places. Now I would like to ask you about sports, fitness and recreational activities<br>अगले प्रश्नों का जवाब देते समय उन सब गतिविधियों को हटा दें जो आप उपर पूछे भारी या मध्यम मेहनत वाली गतिविधियों या कहीं आने जाने के सवालों के जवाब में बता चुके हैं। अब मै आपसे खेल कूद, अपने को चुस्त दरुस्त रखने तथा मनोरंजक क्रीडाओं के बारे में प्रश्न पूछूँगा / पूछूँगी				
P10	Do you do any **vigorous**-intensity **sports**, fitness or recreational (leisure) activities that cause large increases in breathing or heart rate like (running or football or cycling) for at **least 10 minutes continuously**?<br>क्या आप किसी भारी मेहनत वाले खेलों या मनोरंजक गतिविधियों में भाग लेते हैं (भागना, फुटबाल, साइकिल चलाना) जिस से श्वास लेने तथा हृदय गति काफी बढ़ जाती है अथवा जो कम से कम 10 मिनट तक लगातार करते हो<br>Ask the participant to think about recreational vigorous-intensity activities only. Only include recreational activities activities are regarded as vigorous intensity if they cause large increases in breathing and/or heart rate	Yes हाँ .1No.नहीं...2	☐	*If No, go to P13 अगर उत्तरनहीं है तो P13 पर जाएँ*
P11	In a typical week, on how many days do you do vigorous-intensity sports, fitness, or recreational activity?<br>एक सामान्य हफ्ते में आप कितने दिन भारी मेहनत वाले खेलों या मनोरंजक गतिविधियों में भाग लेते हैं?<br>Typical week" means a week when the participant is engaged in his/her usual activities. Valid responses range from 1-7.	Number of days दिनों की संख्या	☐	

Individual ID | | | | | | |

P12	On a typical day that you do any **vigorous**-intensity **sports**, fitness or recreational (leisure) activities how much time do you spend such **vigorous**-intensity activities?<br>एक सामान्य दिन जब आप भारी मेहनत वाले खेलों या मनोरंजक गतिविधियों में भाग लेते हैं, तो उस में कितना समय बिताते हैं ?<br>Ask the participant to think of a typical day he/she can recall easily in which he/she engaged in recreational vigorous-intensity activities. The participant should only consider those activities undertaken continuously for 10 minutes or more. Probe very high responses (over 4 hrs) to verify.	Hours घंटे<br>Minutes मिनट		
P13	Do you do any **moderate**-intensity sports, fitness or recreational (leisure) activities that cause small increases in breathing or heart rate such as brisk walking (cycling, swimming, volleyball etc.) for at least 10 minutes continuously?<br>क्या आप किसी मध्यम मेहनत वाले खेलों या मनोरंजक गतिविधियों में भाग लेते हैं जिस से श्वास लेने तथा ह्रदय गति कुछ बढ़ जाती है अथवा जो कि कम से कम 10 मिनट तक लगातार करते हो जैसे कि तेज चलना, साइकिलिंग, तैराकी, वोलीबॉल खेलना<br>Ask the participant to think about recreational moderate-intensity activities only. Activities are regarded as moderate intensity if they cause small increases in breathing and/or heart rate	Yes हाँ .1<br>No.नहीं...2		*If No, go to P16*<br>*अगर उत्तरनहीं है तो P16 पर जाएँ*
P14	In a typical week, on how many days do you do moderate-intensity sports, fitness, or recreational activities?<br>एक सामान्यहफ्ते में आप कितने दिन मध्यम मेहनत वाले खेलों या मनोरंजक गतिविधियों में भाग लेते हैं?<br>"Typical week" means a week when the participant is engaged in his/her usual activities. Valid responses range from 1-7	Number of days दिनों की संख्या		

Individual ID | | | | | | | |

P15	On a typical day that you do you any **moderate**-intensity sports, fitness, or recreational activities how much time do you spend such **moderate**-intensity activities?<br>एक सामान्य दिन जब आप मध्यम मेहनत वाले खेलों या मनोरंजक गतिविधियों में भाग लेते हैं, तो उस में कितना समय बिताते हैं ?<br>Ask the participant to think of a typical day he/she can recall easily in which he/she engaged in recreational moderate-intensity activities. The participant should only consider those activities undertaken continuously for 10 minutes or more. Probe very high responses (over 4 hrs) to verify.	Hours घंटे<br>Minutes मिनट		
P16	During a **typical or usual day**, how much time do you spend sitting or, reclining and watching television, working on computer, playing game in mobile/tablet , talking with friends, or doing other sitting activities like knitting, embroidery etc? Please also include the time spent sitting in office. **But do NOT include time spent sleeping. एक सामान्य दिन में आप कितना समय बैठ कर या लेट कर टी वी देखने या कंप्यूटर पर काम करने या फ़ोन या टेबलेट पर गेम खेलने, बातचीत करने या अन्य बैठ कर की जाने वाली गतिविधियों जैसे कि सिलाई, कढाई इत्यादिमें बिताते हैं। दफ्तर में बैठे रहने का समय भी इस में जोड़ें।**<br>Ask the participant to consider total time spent sitting at work, in an office, reading, watching television, using a computer, doing hand craftlike knitting, resting etc. The participant should not include time spent sleeping.	Hours घंटे<br>Minutes मिनट		

Yoga				
Now I will ask you about performing yoga				
P17	Do you practice **Yoga (which includes activities like asana, pranayam or mediation)**?<br>क्या आप योग करते हैं (इसमे यह गतिविधियां शामिल हैं : आसन, प्राणायाम,ध्यान)<br>Record the appropriate response	Yes हाँ .1<br>No.नहीं...2		*If No, go to H1*<br>*अगर उत्तरनहीं है तो H1 पर जाएँ*

P18	If yes, how many days in a typical week do you perform the following (complete one row) यदि हाँ तो एक सामान्य हफ्ते में कितने दिन आप यह योग से सम्बंधित गतिविधियाँ करते हैं<br>Valid response for number days 1-7	Number of days in the week	On a usual /typical day that you do yoga , how much time do you spend doing yoga related activities, in case you do the activities in more than one session in a day please add all sessions done in a typical day<br>एक सामान्य दिन जिस दिन आप योग करते हैं उस दिन योग से सम्बंधित गतिविधियों में कितना समय बिताते हैं , पुरे दिन में अगर आप एक से ज्यादा बार योग गतिविधि करते हैं तो उन सब को जोड़ के बताएं		
			Hours	Minutes	
P18a	Asana आसन				
P18b	Pranayam प्राणायाम				
P18c	Meditation ध्यान				

Next I will ask you about some common non communicable disease like hypertension, diabetes, raised cholesterol or cardiovascular diseases. While answering these questions please understand that the term health professional could mean any one related to health system and not necessarily a doctor or a nurse.

History of Raised Blood Pressure

Now I shall ask you about raised blood pressure. अब मै आपसे बढे हुए रक्त चाप के बारे में पूछूँगा ।

**Questions**		**Response**		**Skip**
H1	Have you **ever** had your blood pressure measured by a doctor or other health worker?<br>क्या आपने कभी भी किसी डॉक्टर या स्वास्थ्य कर्मी से अपना रक्त चाप चैक करवाया है ?<br>Ask the participant to only consider measurements done by a doctor or other health worker	Yes हाँ .1No.नहीं...2		*If No, go to H6*

Individual ID

H2	When was your blood pressure last measured by health professional/workers? आखरी बार किसी स्वास्थ्य कर्मी द्वारा आपका रक्त चाप कब मापा गया था<br>Ask him/her to remember and select the response	Within past 6 months ....1<br>Within past 6-12 months.....2<br>Within past 1-2years..........3<br>Not within past 2 years......4		*If not in last 12 months(option 3 or 4 selected), go to H3*
H2a	If measured in last 12 months where was it done?	Government Screening camp ....1<br>Private / NGO Screening camp..2<br>Government health facility..3<br>Private/ NGO health facility..4		
H3	Have you ever been told by a doctor or other health worker that you have raised (high) blood pressure or hypertension? क्या आपको कभी भी किसी डॉक्टर या स्वास्थ्यकर्मी ने बताया हैं कि आपका रक्तचाप बढ़ा हुआ है ?<br>Select the appropriate response.	Yes हाँ .1No.नहीं...2		*If No, go to H6*
H3a	Have you been told so in the past 12 months?<br>क्या ऐसा पिछले १२ महीने में बताया ?<br>Only for those that have previously been diagnosed with raised blood pressure.	Yes हाँ .1No.नहीं...2		
H4	Are you currently consulting any allopathic doctor for hypertension? If yes from which type of health facility	No..1<br>Yes, from Govt.health facility..2<br>Yes, from Pvt/NGO health facility..3		
H4a	In the past 2 weeks on how many days did you take any medication or drugs for raised blood pressure or hypertension that had been prescribed by a doctor or other health worker? पिछले 2 हफ्तों में कितने दिन आपने उच्च रक्त चाप के लिए कोई ऐसी दवाई ली जो की किसी डॉक्टर या स्वास्थ्य कर्मी ने लिखी हो<br>Ask the participant to only consider drugs for raised blood pressure prescribed by a doctor or other health worker and write the number of days if not taken write 00	Number of days दिनों की संख्या		If 00 go to H5

Individual ID | | | | | | | |

H4b	What was the source of the drugs you took for hypertension in last two weeks<br>Multiple choices possible	Govt.Dispensary Yes …1<br>No…2<br>Do Not know..7	☐	
		Chemist/ Private/NGO<br>Dispensary Yes …1<br>No…2<br>Do Not know …7	☐	
H5	Have you ever seen any practitioner from the following systems of medicine for raised blood pressure or hypertension? क्या आप कभी भी इनमें से किसी भी चिकित्सा की प्रणाली के डॉक्टर से उच्च रक्त चाप के लिए मिले<br>Select the appropriate response for each of the following			
H5a	Ayurveda आयुर्वेद	Never….1<br>Yes and currently taking medication…..2<br>Yes but currently not taking medication…..3	☐	
H5b	Siddha सिद्ध	Never…..1<br>Yes and currently taking medication…..2<br>Yes but currently not taking medication….3	☐	
H5c	Unani यूनानी	Never….1<br>Yes and currently taking medication….2<br>Yes but currently not taking medication….3	☐	
H5d	Homeopathy होमियोपैथी	Never….1<br>Yes and currently taking medication….2<br>Yes but currently not taking medication….3	☐	
**History of Diabetes**				
H6	Have you **ever** had your blood sugar been measured by a doctor or health worker?<br>क्या आपने कभी भी किसी डॉक्टर या स्वास्थ्यकर्मी से अपना ब्लड शुगर चैक करवाया है ?<br>Ask the participant to only consider measurements done by a doctor or other health worker.	Yes …1<br>No…2	☐	*If No, go to H12*

H7	When was your blood sugar last measured by health professional/workers? आखिरी बार किसी स्वास्थ्यकर्मी द्वारा आपका ब्लड शुगर कब मापा गया था<br>Ask him/her to remember and select the response	Within past 6 months ....1<br>Within past 6-12 months.....2<br>Within past 1-2years.........3<br>Not within past 2 years......4		
H7a	If measured in last 12 months where was it done?	Government Screening camp .1<br>Private / NGO Screening camp..2<br>Government health facility.3<br>Private/ NGO health facility.4		
H8	Have you ever been told by a doctor or health worker that you have diabetes? क्या आपको कभी भी किसी डॉक्टर या स्वास्थ्यकर्मी ने बताया कि आपको मधुमेह है ?<br>Select the response	Yes ...1<br>No...2		*If No, go to H12*
H8a	Have you been told so in the past 12 months?<br>क्या ऐसा पिछले १२ महीने में बताया ?<br>Only for those that have previously been diagnosed with diabetes	Yes ...1<br>No.....2		
H9	Are you currently consulting any allopathicdoctor for Diabetes? If yes from which type of health facility	No..1<br>Yes, from Govt. health facility..2<br>Yes, from Pvt/NGO health facility..3		
H9a	In the past 2 weeks on how many days did you take any Oral drug s(medication) for raised blood sugar or diabetes prescribed by a doctor or other health worker? पिछले 2 हफ्तों में कितने दिन आपने मधुमेह या बढे हुए ब्लड शुगर के लिए कोई ऐसी दवाई (मुह से खाने वाली) ली जो की किसी डॉक्टर या स्वास्थ्य कर्मी ने लिखी हो<br>Ask the participant to only consider drugs for diabetes prescribed by a doctor or other health worker	Number of days दिनों की संख्या		

Individual ID | | | | | | | |

H10	In the past 2 weeks on how many days did you take Insulin for raised blood sugar or diabetes prescribed by a doctor or other health worker? पिछले 2 हफ्तों में कितने दिन आपने मधुमेह या बढे हुए ब्लड शुगर के लिए इन्सुलिन ली जो की किसी डॉक्टर या स्वास्थ्य कर्मी ने लिखी हो<br>Ask the participant to only consider insulin that was prescribed by a doctor or other health worker	Number of days दिनों की संख्या		If 00 go to H11
H10b	What was the source of the drugs you took for Diabetes in last two weeks<br>Multiple choices possible	Govt.Dispensary Yes …1<br>No…2<br>Do not Know..7		
		Chemist/ Private/NGO Dispensary Yes …1<br>No…2<br>Do not Know..7		
H11	Have you ever seen any practitioner of the following systems of medicine for raised blood sugar or diabetes? क्या आप कभी भी इनमें से किसी भी चिकित्सा की प्रणाली के डॉक्टर से मधुमेह या बढे हुए ब्लड शुगर के लिए मिले<br>Select the appropriate response for each of the following			
H11a	Ayurveda आयुर्वेद	Never…कभी नहीं ..1<br>Yes and currently taking medication हाँ दवाई भी ले रहे हैं ..2<br>Yes but currently not taking medication हाँ पर अभी दवाई नही ले रहे .3		
H11b	Siddha सिद्ध	Never…कभी नहीं ..1<br>Yes and currently taking medication हाँ दवाई भी ले रहे हैं ..2<br>Yes but currently not taking medication हाँ पर अभी दवाई नही ले रहे .3		
H11c	Unani यूनानी	Never…कभी नहीं ..1<br>Yes and currently taking medication हाँ दवाई भी ले रहे हैं ..2<br>Yes but currently not taking medication हाँ पर अभी दवाई नही ले रहे .3		
H11d	Homeopathy होमियोपैथी	Never…कभी नहीं ..1<br>Yes and currently taking medication हाँ दवाई भी ले रहे हैं ..2<br>Yes but currently not taking medication हाँ पर अभी दवाई नही ले रहे .3		
**History of raised total cholesterol**				

Individual ID | | | | | | | |

H12	Have you had your cholesterol (fat levels in your blood) measured by a doctor or other health worker? क्या आपने कभी भी किसी डॉक्टर या स्वास्थ्यकर्मी से अपना कालेस्ट्राल (खून में वसा की मात्रा)चैक करवाया है ?<br>Ask the participant to only consider measurements done by a doctor or other health worker	Yes ......1<br>No 2		*If No, go to H16*
H13a	Have you ever been told by a doctor or other health worker that you have raised cholesterol?<br>क्या आपको कभी भी किसी डॉक्टर या स्वास्थ्यकर्मी ने आपको बताया कि आपका कालेस्ट्राल है बढ़ा हुआ है ?<br>Select the response. Ask him/her to remember any visit to a doctor or other health worker.	Yes .....1<br>No.... 2		*If No, go to H16*
H13b	Have you been told so in the past 12 months?<br>क्या ऐसा पिछले १२ महीने में बताया ?<br>Only for those that have previously been diagnosed with raised total cholesterol.	Yes ...1<br>No.....2		
H14	In the past 2 weeks on how many days did you take any Oral drugs (medication) for raised cholesterol prescribed by a doctor or other health worker? पिछले 2 हफ्तों में कितने दिन आपने बढ़े कालेस्ट्राल के लिए कोई ऐसी दवाई (मुह से खाने वाली) ली जो की किसी डॉक्टर या स्वास्थ्य कर्मी ने लिखी हो<br>Ask the participant to only consider drugs for cholesterol prescribed by a doctor or other health worker	Number of days दिनों की संख्या		If 00 go to H15
H14b	What was the source of the drugs you took for lowering cholesterol in last two weeks<br>Multiple choices possible	Govt.Dispensary Yes ...1<br>No...2<br>Do not Know..7		
		Chemist/ Private/NGO Dispensary Yes ...1<br>No...2<br>Do not Know..7		

Individual ID | | | | | | | |

H15	Have you ever seen any practitioner from the following systems of medicine for raised cholesterol?<br>आप कभी भी इनमें से किसी भी चिकित्सा की प्रणाली के डॉक्टर से बढे हुए कालेस्ट्राल के लिए मिले हैं ?<br>Select the appropriate response for each of the following.			
H15a	Ayurveda<br>आयुर्वेद	Never...कभी नहीं ..1<br>Yes and currently taking medication हाँ दवाई भी ले रहे हैं ..2<br>Yes but currently not taking medication हाँ पर अभी दवाई नही ले रहे .3	☐	
H15b	Siddha सिद्ध	Never...कभी नहीं ..1<br>Yes and currently taking medication हाँ दवाई भी ले रहे हैं ..2<br>Yes but currently not taking medication हाँ पर अभी दवाई नही ले रहे .3	☐	
H15c	Unani यूनानी	Never...कभी नहीं ..1<br>Yes and currently taking medication हाँ दवाई भी ले रहे हैं ..2<br>Yes but currently not taking medication हाँ पर अभी दवाई नही ले रहे .3	☐	
H15d	Homeopathy<br>होमियोपैथी	Never...कभी नहीं ..1<br>Yes and currently taking medication हाँ दवाई भी ले रहे हैं ..2<br>Yes but currently not taking medication हाँ पर अभी दवाई नही ले रहे .3	☐	

**History of Cardiovascular Diseases Cerebro-Vascular Accident**				
H16	Have you ever had chest pain (heart related)or a heart attack (angina) or a stroke (cerebro-vascular accident or incident) that was diagnosed in a hospital?<br>क्या कभी भी आपको छाती में दर्द (ह्रदय रोग की वजह से) हुआ या किसी हस्पताल में हार्ट अटैक या घात या सेरिब्रो वास्कुलर इवेंट का निदान हुआ<br>Select the appropriate response.	Yes.हाँ ...1<br>No नहीं ...2<br>Don't Know...मालूम नहीं .7<br>No response/refuse..8	☐	If No or don't know go to H17
H16a	If yes at what type of health facility was it diagnosed	Govt. health facility..1<br>Pvt/NGO health facility..2	☐	
H17	In the last 2 weeks on how many days did you take aspirin to prevent or treat heart disease or stroke? पिछले 2 हफ्तों में कितने दिन आपने ह्रदय रोग या घात होने को रोकने के लिए एस्पिरिन ली ?	Number of days दिनों की संख्या	☐☐	

Individual ID | | | | | | | |

H18	Are you currently taking statins (Lovastatin /Simvastatin/Atorvastatin or any other statin) or any other cholesterol lowering drugs regularly to prevent or treat heart disease or stroke? क्या आप आजकल हृदय रोग या घात होने को रोकने के लिए स्टेटइन नामक दवाई समूह में से कोई दवाई (लोवा स्टेटइन सिम्वुस्टेटइन अटोरवास्टेटइन इतियादी) या अन्य कोलेस्ट्रॉल लोकम करने की दवाई ले रहे हैं ?<br>"Regularly" means on a daily or almost daily basis	Yes हाँ ...1<br>No नहीं 2	☐	

**Lifestyle Advice**				
H19	During the past one year, has a doctor or other health worker advised any of the following?क्या पिछले एक साल में किसी डॉक्टर या स्वास्थ्यकर्मी ने आपको इन में से कोई सलाह दी है ?*Please Record for each*<br>Select the appropriate response. Ask the participant to only consider advice from a doctor or other health worker			
H19a	Against starting smoking tobacco or quitting smoking धुम्रपान न करने या धुम्रपान बंद करने की सलाह	Yes ...1<br>No ...2<br>Did not meet any doctor or health worker =3	☐	If did not meet any health worker or doctor, GO TO Cancer Screening section
H19b	Against starting use of smokeless tobacco or quitting smokeless tobacco धूम्र रहित तम्बाकू सेवन शुरू न करने या धूम्र रहित तम्बाकू सेवन बंद करने की सलाह	Yes ...1<br>No ...2<br>Did not meet any doctor or health worker =3	☐	
H19c	Against starting taking alcohol or decrease intake or alcohol cessation शराब सेवन शुरू न करने या शराब सेवन बंद करने की सलाह	Yes ...1<br>No ...2<br>Did not meet any doctor or health worker =3	☐	
H19d	Reduce salt in your diet अपने खाने में नमक की मात्रा कम करने की सलाह	Yes ...1<br>No ...2<br>Did not meet any doctor or health worker =3	☐	

Individual ID | | | | | | |

H19e	Increase fruit and vegetable intake in your diet अपने खाने में फल अथवा सब्जियों के सेवन को बढाने की सलाह	Yes ...1<br>No ...2<br>Did not meet any doctor or health worker =3		
H19f	Reduce fat in your diet अपने खाने में वसा की मात्र कम करने की सलाह	Yes ...1<br>No ...2<br>Did not meet any doctor or health worker =3		
H19g	Increase physical activity अपनी शारीरिक क्रियाशीलता बढ़ाने की सलाह	Yes ...1<br>No ...2<br>Did not meet any doctor or health worker =3		
H19h	Maintain a healthy body weight or lose weight अपना वजन स्थायी रखने या कम करने की सलाह	Yes ...1<br>No ...2<br>Did not meet any doctor or health worker =3		
H19i	Consume special prescribed diet कोई ख़ास आहार खाने की सलाह	Yes ...1<br>No ...2<br>Did not meet any doctor or health worker =3		
H19j	Practice Yoga योग करने की सलाह	Yes ...1<br>No ...2<br>Did not meet any doctor or health worker =3		

Cancer Screening (To be administered to those above 30 years of age only)

Certain examination or tests are done to look for cancer in its early stage and this is known as cancer screening. Different cancers have different screening methods. You may have been subjected to any of the methods. So please try to recall if any of the methods I shall be describing below in the relevant section were done for you. कैंसर के जल्द निदान के लिए कुछ जांचे की जाती हैं इनको कैंसर के लिए स्क्रीनिंग कहा जाता है इसके अलग अलग तरीके होते हैं अब मै आपको कुछ तरीकों के बारे में बताऊंगा आप याद करने की कोशिश करें की क्या कभी इस तरह की आपकी जांच हुई है ?

Read this opening statement out loud. It should not be omitted.

	**Oral cancer screening**			
CX1	Have you ever had any doctor or dentist examined your oral cavity to look for **early signs of cancer**?क्या कभी भी किसी डाक्टर या दन्त चिकित्सक ने कैंसर के लक्षणों के लिए आपके मुहं की जांच की है	Yes ...1<br>No ...2<br>Don't know...7		*If respondent is a Male go to Section 2 M1*

Individual ID | | | | | | | |

	Remember that it does not include oral examination for a dental problem or other non-cancer related problems.	No response/ refused..8		
	**Breast Cancer screening** (For Women only)			
CX2	Have you ever performed Breast Self-Examination to look for breast cancer? क्या आपने कभी स्तन कैंसर के लिए स्वयं जाँच की है Select the appropriate response	Yes… 1<br>No… 2	☐	
CX3	Have you ever had any breast examination by a doctor to look for Breast Cancer? क्या कभी किसी डाक्टर ने स्तन कैंसर के लिए आपकी जाँच की है Select the appropriate response. Ask the participant for any breast examination being done on her by a doctor.	Yes… 1<br>No… 2	☐	
CX4	Have you ever undergone an ultrasound of breast or mammography? क्या आपके स्तनो की कभी भी अल्ट्रासोऊड या मैम्मोगराफी हुई है Select the appropriate response. Ask the participant for any ultrasound breast of mammography being done.	Yes …1<br>No …2	☐	
	**Cervical Cancer Screening** (For Women only)			

Following questions are for cervical cancer prevention. Screening tests for cervical cancer prevention can be done in different ways like Visual Inspection with Acetic Acid/vinegar (VIA), pap smear & Human Papillo mavirus (HPV) test.

अगले कुछ प्रश्न बच्चेदानी के मुंह के कैंसर की रोकथाम से सम्बंधित हैं | इस के लिए विभिन्न प्रकार की स्क्रीनिंग जांच की जा सकती हैं जैसे की एसिटिक एसिड लगा के बच्चेदानी के मुंहरंग के बदलाव की जींच या फिर पैप स्मीयर या एच पी वी जाँच जिसमे नर्स या डॉक्टर एक रुई की फाँस को योनी के अंदर छुआ के जाँच के लिए नमूना लेते हैं यह भी हो सकता है की आपको स्वयं फाँस छुआ कर देने को कहा जाये | यह जांचे लैब में भेज कर कोशिकाओं में असामान्य बदलाव या एच पी वाईरस की मौजूदगी का पता लगाती हैं

- VIA is an inspection of the surface of the uterine cervix after acetic acid (or vinegar) has been applied to it.
- For both pap smear & HPV test, a doctor / nurse uses a swab to wipe from inside your vagina, take a sample and send it to a laboratory.

Individual ID | | | | | | |

• It is even possible that you were given the swab yourself and asked to swab the inside of your vagina. The laboratory checks for abnormal cell changes if a pap smear is done and for the HP virus if an HPV test is done.<br>Read this opening statement out loud. It should not be omitted				
CX5	Have you ever had a screening test for cervical cancer, using any of these methods described above? क्या जो तरीके बताये गए उन में से किसी से भी आपके बच्चेदानी के मुंह के कैंसर के लिए जांच हुई है ?<br>Remember that it includes both self-sought as well as opportunistic screening for cervical cancer.	Yes… 1<br>No… 2<br>Don't know …7<br>No response/ refused..8	☐	

Individual ID | | | | | | | |

Section 2. Physical Measurement:				
M0	Are you willing to undergo anthropometric measurements?	Yes =1<br>No=2		If no go to Z1
M1	(For Women) Are you pregnant?<br>केवल महिलाओं को पूछें : क्या आप गर्भवती हैं ?<br>Pregnant women skip over height, weight, waist and hip measurements.	Yes ......1<br>No 2		**If yes measure only her BP mark 9 in all other boxes and end interview**
M2	**Is the respondent bedridden or, missing a limb or multiple limbs?**<br><br>**If yes Specify**<br><br>________________<br><br>If yes Specify and accordingly skip the measurement which cannot be done e.g. in case both upper limbs are missing cannot take BP. In case of absent limbs weight would be lesser than usual	Yes ......1<br>No 2		*If the respondent is invalid (both arms missing and BP cannot be measured or due to paraplegia cannot stand up and his /her height cannot be measured due to this reason write 666.6*

Blood Pressure and Pulse Rate

- *For blood pressure (BP) measurement the respondent must be comfortable and if the respondent had been exerting then he/she must rest for at least 5 minutes.*
- *There must be at least 5 minutes gap between two consecutive readings.*
- *If the respondent refuses any of the measurement write 88or 888*

	Physical Measurement	Response	
M3	Technician ID<br>Record technician ID (in most cases interviewer would be the same		| | | | |

	as for behavioural measurements).		
M4	Enter last two digits of the Device ID for Blood Pressure<br>Record device ID.		
M4a	B.P. Reading 1<br>➢ *If cannot measure due to handicap mark 666.6*	Systolic (mmHg)	
M4b	Record first measurement after the participant has rested for 15 minutes. Wait 3 minutes before taking second measurement.	Diastolic (mmHg)	
M4c	Pulse Rate Reading 1<br>Record the reading	Beats/min	
M5a	B.P. Reading 2<br>➢ *If cannot measure due to handicap mark 666.6*	Systolic (mmHg)	
M5b	Record first measurement after the participant has rested for 15 minutes. Wait 3 minutes before taking second measurement	Diastolic (mmHg)	
M5c	Pulse Rate Reading 2<br>Record the reading	Beats/min	
M6a	B. P. Reading 3<br>➢ *If cannot measure due to handicap mark 666.6*	Systolic (mmHg)	
M6b	Record third measurement.	Diastolic (mmHg)	
M6c	Pulse Rate Reading 3<br>Record the reading	Beats/min	
**Height, weight and waist**			
M7	Technician / Interviewer ID<br>Record interviewer ID (in most cases interviewer would be the		

Individual ID ☐☐☐☐☐☐☐

	same as for behavioural and blood pressure measurements)		
M8a<br><br><br>M8b	Enter last two digits ofthe Device ID for height and weight<br>Record device IDs	Height.......<br><br><br>Weight......	☐☐<br>☐☐
M9	Height<br>➢ *If Pregnantmark 999.9*<br>➢ *If cannot measure due to handicap mark 666.6*<br>Record participant's height in cm with one decimal point.	In Centimeter (cm)...	☐☐☐.☐
M10	Weight<br>➢ *If Pregnantmark 999.9*<br>➢ *If cannot measure due to handicap mark 666.6*<br>➢ *If too large for scale 666*<br>Record participant's weight in kg with one decimal point	In Kilograms (kg)...	☐☐☐.☐
M11	Enter last two digits of the Device ID for waist<br>Record device ID.		☐☐
M12	Waist circumference<br>➢ *If Pregnantmark 999.9*<br>➢ *If cannot measure due to handicap mark 666.6*<br>Record participant's waist circumference in centimeters with one decimal point	In Centimeter(cm)	☐☐☐.☐

Individual ID | | | | | | | |

	**Location**			
Z1	Record GPS coordinates			
	No GPS coordinates entered. Please try again.			
Z2	Number of visits made	One……………….1<br>Two……………….2<br>Three…………….3		
Z3	Outcome	Refused not filled ………….1<br>Locked not filled……………2<br>Form incomplete due to midway refusal …..3<br>Form incomplete as person had to leave midway………………….4<br>Form complete…..5<br>Form incomplete need to revisit….6		

Section 3. Biochemical Measurement:				
BC1	Did participant provide blood for blood sugar measurement	Yes …1<br>No…..2	☐	**If no go to BC 10**
BC2	During the past 12 hours have you had anything to eat or drink, other than water?	Yes …1<br>No…..2	☐	
BC3	Date of last meal		☐☐☐☐☐☐	
BC4	Time of last meal		☐☐ : ☐☐	
BC5	If you are a diabetic, have you taken insulin or other drugs (medication) that have been prescribed by a doctor or other health worker for raised blood glucose today?	Not a known Diabetic…1<br>Yes, taken …2<br>No, not taken …..3	☐	
BC6	Technician ID		☐☐☐☐	
BC7	Enter the last two digits of the device ID		☐☐	
BC8	Time of testing		☐☐ : ☐☐	
BC 9	Fasting blood sugar (mg/dl)		☐☐☐	
BC 10	Did participant provide urine sample?	Yes …1<br>No…..2	☐	**If no go to Z2**
BC 11	Had you been fasting prior to the urine collection?	Yes …1<br>No…..2	☐	
BC 12	Date of urine collection		☐☐☐☐☐☐	
BC 13	Time of day urine sample taken (24 hour clock)		☐☐ : ☐☐	
BC 14	Technician ID		☐☐☐☐	
BC 15	Amount of urine brought to the camp (in ml)		☐☐	
BC 16	Number of spot urine aliquots made		None =1<br>One =2<br>Two =3	if none go to BC17 else go to outcome
BC 17	PROVIDE REASON for not making any urine aliquots		____________________	

Z2	Number of attempts madeto collect blood sugar / urine samples	One……………….1<br>Two……………….2<br>Three…………….3		
Z3	Outcome	Refused not tested…………1<br>Did not come to camp not tested……………2<br>Blood sugar tested Urine sample not provided..3<br>Blood sugar not tested, urine samples collected…4<br>Both Blood sugar and Urine samples collected……………….5<br>Not collected will attempt again…..6		
