# Supplementary material for: National noncommunicable disease monitoring survey (NNMS) in India: Estimating risk factor prevalence in adult population
Source: PLoS One. 2021 Mar 2;16(3):e0246712. doi: 10.1371/journal.pone.0246712 (PMC7924800; doi:10.1371/journal.pone.0246712)
Supplement: S1 Table — (DOCX) [file pone.0246712.s002.docx]

**S1 Table. NCD risk factors (behavioural and metabolic) among adults (18-69 years)**

| **Behavioral risk factors (18-69 years)** | **Men** | **Women** |  | **Urban** | **Rural** |  | **Overall**  % (95% CI) |
| --- | --- | --- | --- | --- | --- | --- | --- |
|  | % (95% CI) | % (95% CI) |  | % (95% CI) | % (95% CI) |  |  |
| **Tobacco use** | | | | | | | |
| Daily tobacco use  (Smoked or smokeless tobacco) | 43.2  (40.4-46.1) | 11.4  (9.7-13.5) |  | 20.5  (17.0-24.4) | 31.7  (29.5-34.0) |  | 28.0  (26.0-30.0) |
| Daily smoked tobacco use | 17.5  (15.5-19.7) | 1.2  (0.8-1.8) |  | 8.7  (6.5-11.5) | 10.1  (8.8-11.7) |  | 9.7  (8.5-11.0) |
| Bidi | 13.6  (11.9-15.5) | 0.7  (0.4-1.1) |  | 4.9  (4.0-6.1) | 8.6  (7.4-10.1) |  | 7.4  (6.5-8.4) |
| Cigarette | 4.5  (3.4-6.0) | 0.2  (0.1-0.9) |  | 4.2  (2.6-6.9) | 1.6  1.1-2.2) |  | 2.5  (1.8-3.4) |
| Hookah / shisha | 0.4  (0.2-1.0) | 0.03  (0.006-0.2) |  | 0.01  (0.002-0.1) | 0.3  (6.5-8.4) |  | 0.2  (0.1-0.5) |
| Daily smokeless tobacco use | 30.6  (28.0-33.4) | 10.5  (8.8-12.5) |  | 14.4  (11.6-17.6) | 24.3  (22.1-26.7) |  | 21.0  (19.1-22.9) |
| Daily smoked and smokeless tobacco use | 4.9  (3.9-6.2) | 0.2  (0.1-0.7) |  | 2.6  (1.5-4.6) | 2.7  (2.1-3.4) |  | 2.7  (2.1-3.4) |
| **Alcohol use** | | | | | | | |
| Ever Consumed Alcohol | 33.9  (31.1-36.8) | 2.7  (1.7-4.2) |  | 17.2  (15.2-19.3) | 19.8  (17.3-22.5) |  | 18.9  (17.1-20.8) |
| Consumed in past 30 days | 22.6  (20.4-24.9) | 1.8  (1.0-3.2) |  | 11.0  (9.7-12.5) | 13.3  (11.3-15.7) |  | 12.6  (11.1-14.2) |
| Amongst ever users, those who abstained in past 12 months | 16.4  (14.1-19.0) | 9.4  (4.6-18.2) |  | 17.3  (13.7-21.6) | 15.3  (12.5-18.6) |  | 15.9  (13.6-18.5) |
| **Dietary practices** | | | | | | | |
| Often / always add extra salt right before eating | 16.4  (14.4-18.6) | 13.7  (11.8-15.8) |  | 13.1  (10.5-16.2) | 16.1  (14.0-18.4) |  | 15.1  (13.4-16.9) |
| Think that lowering salt consumption is important | 69.7  (14.0-18.4) | 58.4  (55.1-61.7) |  | 71.6  (67.7-75.2) | 60.6  (57.0-64.1) |  | 64.3  (61.6-66.9) |
| Took steps to reduce salt intake | 48.3  (44.6-52.1) | 42.1  (38.2-46.2) |  | 47.9  (42.8-52.9) | 44.1  (39.6-48.7) |  | 45.4  (42.0-48.8) |
| **Physical activity** | | | | | | | |
| Voluntary physical activity | 17.2  (15.3-19.2) | 3.4  (2.7-4.2) |  | 14.8  (12.6-17.3) | 8.3  (7.1-9.8) |  | 10.5  (9.4-11.7) |
| **Metabolic risk factors (18-69 years)** | | | | | | | |
| Central obesity | 24.4  (22.1-26.9) | 40.7  (37.8-43.7) |  | 48.2  (44.9-51.5) | 24.2  (21.7-26.9) |  | 32.2  (29.9-34.5) |
