# Supplementary material for: National noncommunicable disease monitoring survey (NNMS) in India: Estimating risk factor prevalence in adult population
Source: PLoS One. 2021 Mar 2;16(3):e0246712. doi: 10.1371/journal.pone.0246712 (PMC7924800; doi:10.1371/journal.pone.0246712)
Supplement: S2 Table — (DOCX) [file pone.0246712.s003.docx]

**S2 Table. NCD risk factors among adults aged between 18-69 years (Mean)**

| **Risk factors (18-69 years) in Mean** | **Men**  (95% CI) | **Women**  (95% CI) |  | **Urban**  (95% CI) | **Rural**  (95% CI) |  | **Overall**  (95%CI) |
| --- | --- | --- | --- | --- | --- | --- | --- |
| **Dietary practices** | | | | | | | |
| Mean servings of fruits and / or vegetables per day | 1.8  (1.7-1.9) | 1.6  (1.5-1.7) |  | 1.9  (1.8-2.1) | 1.6  (1.5-1.7) |  | 1.7  (1.6-1.8) |
| **Physical activity** | | | | | | | |
| Minutes spent being sedentary in a day | 290.5  (276.9-304.2) | 328.5  (314.5-342.4) |  | 324.4  (304.8-343.9) | 301.1  (285.3-316.9) |  | 308.9  (296.6-321.2) |
| Total minutes spent in physical activity per day | 118.8  (109.9-127.7) | 55.3  (49.4-61.2) |  | 62.2  (54.2-70.2) | 101.1  (93.5-108.7) |  | 88.1  (82.0-94.1) |
| Body Mass Index (Kg/m^2^) | 22.3  (22.0-22.5) | 22.8  (22.5-23.1) |  | 24.3  (24.1-24.6) | 21.6  (21.4-21.9) |  | 22.5  (22.3-22.8) |
| Mean systolic blood pressure (mm Hg) | 126.1  (125.3-127.0) | 121.8  (121.0-122.7) |  | 126.7  (125.6-127.8) | 122.7  (121.9-123.5) |  | 124.1  (123.4-124.7) |
| Mean diastolic blood pressure (mm Hg) | 81.8  (81.2-82.3) | 80.1  (79.6-80.5) |  | 82.7  (82.1-83.3) | 80.1  (79.5-80.6) |  | 80.9  (80.5-81.4) |
| Mean fasting blood glucose (mg/dl) | 95.1  (93.6-96.6) | 98.5  (96.9-100.1) |  | 101.6  (98.7-104.5) | 94.4  (93.0-95.9) |  | 96.7  (95.3-98.1) |
